# Development of a Week-Long Mathematics Intervention for Incoming Chemistry Graduate Students

**DOI:** 10.1021/acs.jchemed.2c00915

**Published:** 2023-08-24

**Authors:** Rachel Clune, Avishek Das, Dipti Jasrasaria, Elliot Rossomme, Orion Cohen, Anne M. Baranger

**Affiliations:** ‡ Kenneth S. Pitzer Center for Theoretical Chemistry, University of California, Berkeley, California 94720, United States; ¶ Department of Chemistry, University of California, Berkeley, California 94720, United States; § Chemical Sciences Division, Lawrence Berkeley National Laboratory, Berkeley, California 94720, United States; ∥ Materials Science Division, Lawrence Berkeley National Laboratory, Berkeley, California 94720, United States; ⊥ Graduate Group in Science and Mathematics Education, University of California, Berkeley, California 94720, United States

**Keywords:** mathematics, intervention, inequity, physical chemistry, graduate education, active learning, group learning, sense of belonging

## Abstract

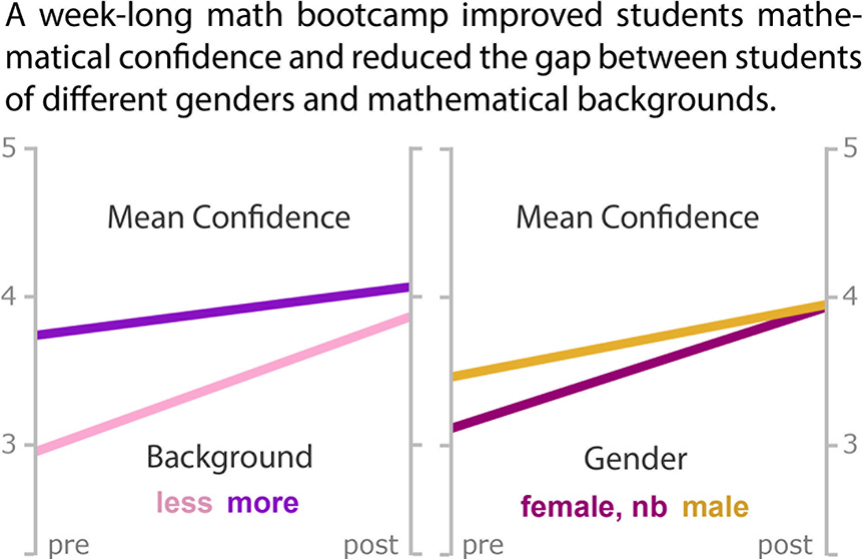

A student-led mathematics bootcamp has been designed and implemented to help foster community building, improve confidence in mathematical skills, and provide mathematical resources for incoming physical chemistry doctoral students. The bootcamp is held immediately before the start of the first semester of graduate school and uses an active learning approach to review and practice undergraduate-level mathematics problems over 5 days in small student groups. This work includes the development and presentation of a new, publicly available mathematics curriculum for the bootcamp on select mathematics topics, including calculus, linear algebra, functions, differential equations, statistics, and coding in Python, aiming at improving students’ confidence and learning experiences in graduate quantum mechanics and statistical physics courses. Surveys before and after the bootcamp showed an increase in students’ confidence in problem-solving in key mathematical areas and social aspects of peer-led group learning. Qualitative and quantitative analyses demonstrate that the bootcamp reduced prior inequities in students’ confidence metrics based on gender and mathematical background.

## Introduction

First-semester coursework for incoming physical chemistry doctoral students at UC Berkeley typically consists of courses in quantum mechanics and statistical mechanics, and these courses represent one of the most challenging aspects of the beginning of graduate school. These fast-paced courses assume conceptual knowledge of and computational skills in a wide range of math topics including multivariable calculus, linear algebra, and probability and statistics. Chemistry graduate programs typically do not teach prerequisite mathematics courses, and incoming students are often unfamiliar with these topics due to differences in their undergraduate math requirements. Furthermore, in the absence of a mathematics coursework plan, students may have difficulty anticipating their own gaps in mathematical knowledge and identifying resources that could address these knowledge gaps. Additionally, Berkeley’s graduate courses encourage students to work together to tackle material and solve homework problems. A critical analysis of barriers to social inclusion in education suggests that the dynamics of finding comfortable working groups in the first few weeks of the semester may exacerbate existing inequities in confidence among students minoritized through race, ethnicity, and gender.^1,2^

The challenges outlined above can lead to poor performance in first-semester courses, which may in turn negatively impact students’ self-concepts of scientific ability, heighten their perceptions of imposter syndrome, and decrease their sense of belonging in the Chemistry Ph.D. program and the department as a whole.^3−5^ In order to address the difficulties students face at the start of their graduate education and in the absence of a more institutional change in mathematics pedagogy for graduate students in their first year, the authors developed and implemented a week-long “bootcamp” intervention for incoming graduate students in the UC Berkeley College of Chemistry. This work reports on the structure and methods of this bootcamp as well as its impact on the incoming chemistry graduate students.

Educational discrepancies like those outlined above reflect broad trends in the effect of mathematics preparedness on student achievement in the fields of science, technology, engineering, and mathematics (STEM).^6−8^ While one might anticipate that mathematics competency plays a dominant factor in student trajectories in STEM fields, a significant body of research indicates that student confidence or self-efficacy also plays a determining role in further engagement with mathematics-related disciplines.^9,10^ These self-efficacy gaps have been documented as early as elementary school,^11^ and they are predictive of engagement in mathematics and related disciplines later in life.^12^ In the context of graduate education in STEM, students with above-average math proficiency^13^ and high degrees of mathematics self-efficacy^14,15^ are more likely to make an initial decision to attend graduate school and to persist through the graduate program.

The crucial role that self-efficacy plays in graduate-level participation in STEM fields highlights a structural challenge in diversifying the academy. Specifically, existing inequities that stigmatize the involvement of students belonging to minoritized groups in STEM fields can result in their attrition from the pipeline of math-intensive disciplines. Many existing frameworks of mathematical education do not account for disparities in prior educational opportunities for minoritized students, thus amplifying existing inequities.^16,17^ Additionally, biased perceptions of student ability and performance discourage underrepresented students from pursuing further mathematics.^14,15,17^ Even programs explicitly designed to improve math preparedness for all high school students, including those from historically underrepresented groups, have been shown to inadvertently increase achievement gaps and negatively affect progression through the pipeline of STEM coursework.^18^

These examples highlight the importance of adopting a critical feminist and race theoretic framework to challenge the disparity in mathematics self-efficacy in minoritized students.^14,19,20^ The word *critical* in this context refers to a pedagogical framework that is self-reflective about the racial, ethnic, and gender-based systems of power it perpetuates.^21^ The development of mathematics curricula that improve student self-efficacy, learning, and retention, while also directly acknowledging and addressing the frameworks of injustice that lead to unequal outcomes in learning and sense of belonging,^22−24^ is therefore critical for diversity, equity, and inclusion (DEI) initiatives.

One class of approaches to create equitable education is represented by innovative pedagogies.^25−34^ While the effectiveness of these pedagogical strategies for chemistry graduate students, to the best of our knowledge, has not been explored, many of these strategies have been developed to improve learning outcomes in the context of undergraduate-level chemistry. Among these is process-oriented guided inquiry learning (POGIL), in which students engage with new concepts via a cycle of learning that consists of exploration, concept invention, and then application.^25^ POGIL has been shown to increase positive attitudes toward chemistry and self-efficacy^26^ as well as decrease the number of alternate (and inaccurate) conceptions held by students across different demographics.^27^ So-called flipped classroom models have also been developed to improve student educational performance. In contrast to POGIL, which emphasizes self-directed learning during class time, these methods encourage students to learn the content outside of class.^28,29^ Adoption of flipped classroom models has shown to improve short-term learning outcomes;^30^ however, it has also led to lower levels of long-term understanding and widened achievement gaps between white male students and their peers.^30^ These effects may be due to the demands of this pedagogical model with regards to required work outside of class hours, which may disadvantage students from certain backgrounds and those with time constraints.^29^ However, when combined with POGIL, a flipped classroom general chemistry course was shown to increase the number of passing grades in a cohort primarily composed of students from underrepresented racial groups.^31^ Finally, peer-led team learning (PLTL) has also proven successful in achieving equitable student outcomes. In this approach, previously successful students lead workshops that review the material and problem-solving strategies. PLTL has been shown to improve student attitudes and performance and is now widely adopted across general chemistry programs at the college level as well as in other STEM disciplines.^32−34^

Each of these three pedagogies can be categorized as active learning and are grounded in constructivist theory.^35−38^ Notably, active learning approaches improve student exam scores and passing rates for all students and narrow the achievement gap for minoritized students across STEM fields.^39^ Within such frameworks, the learning process is understood to be aided by facilitating communication between peers and between instructors and students. Specifically, an integral part of PLTL is the sharing of individual student experiences as anecdotal stories, which can challenge hegemonic assumptions about the composition and background of the student body.^40^ However, the dynamics of autonomous group formation among students in PLTL can sometimes exacerbate the exclusion of minoritized students. Thus, a conscious effort must be made to share authority among students through transparent and flexible role assignments.^2,41^ Even outside of traditional science classrooms, such as those for education and nursing, the use of problem-based learning has been shown to align with feminist pedagogical practices.^42^ While ultimately intended for implementation in long-term educational settings, active learning principles could reasonably improve student outcomes in the context of short-term interventions, such as bootcamps. Literature precedent concerning the effectiveness of mathematics bootcamps for chemistry students is sparse, but similar interventions have been found to increase both knowledge and self-efficacy for graduate students in other disciplines like statistics,^43^ biology,^44^ and economics and political sciences.^45,46^

In addition to the preceding body of literature, the collective experiences of the authors first as physical chemistry graduate students and then as graduate chemistry instructors made evident the need for a mathematics intervention for incoming graduate students. Thus, the authors designed, wrote, and taught the curriculum for a one-week-long math bootcamp aimed at improving physical chemistry graduate students’ ability and confidence in solving undergraduate-level mathematics problems relevant to their quantum mechanics and statistical mechanics coursework. As outlined in the next section, the bootcamp was designed by using principles centered in active learning and critical theory to serve a diverse cohort of participants. This bootcamp provides students with vetted resources, summarized notes, and math practice problems, while encouraging them to work together on problem-solving in a low-stakes, nonjudgmental environment led by peers.

The impact of the bootcamp on achieving the aforementioned goals is evaluated and discussed through the analysis of qualitative and quantitative data and narrative quotes obtained from students and the instructors of their first-year graduate coursework. The methods of data collection, which emphasizes student experiences, and interpretation are aligned with a critical equity-centered framework.^2,22,23^ Overall, this week-long bootcamp boosts student self-efficacy in mathematics, increases student confidence in asking for help from peers and instructors, and decreases gaps in these metrics for students from underrepresented groups. Additionally, this work presents one of the first evaluations of short-term interventions based on active learning approaches for chemistry students at the graduate level.

## Bootcamp Content and Structure

Incoming graduate students in physical chemistry at UC Berkeley were the primary target audience of our bootcamp. The mathematical content was chosen to prepare students for introductory graduate coursework in both quantum and statistical mechanics and to empower students to tackle unanticipated mathematical challenges in the classroom. Addressing mathematical barriers to success was one of the main focuses of this work, and content in physics and chemistry was intentionally omitted. Consistent with the centering of student experiences in critical theory, all aspects of the structure of the bootcamp were informed by the authors’ experiences taking and, in some cases, teaching this introductory series of coursework as student instructors.

**Figure 1:**
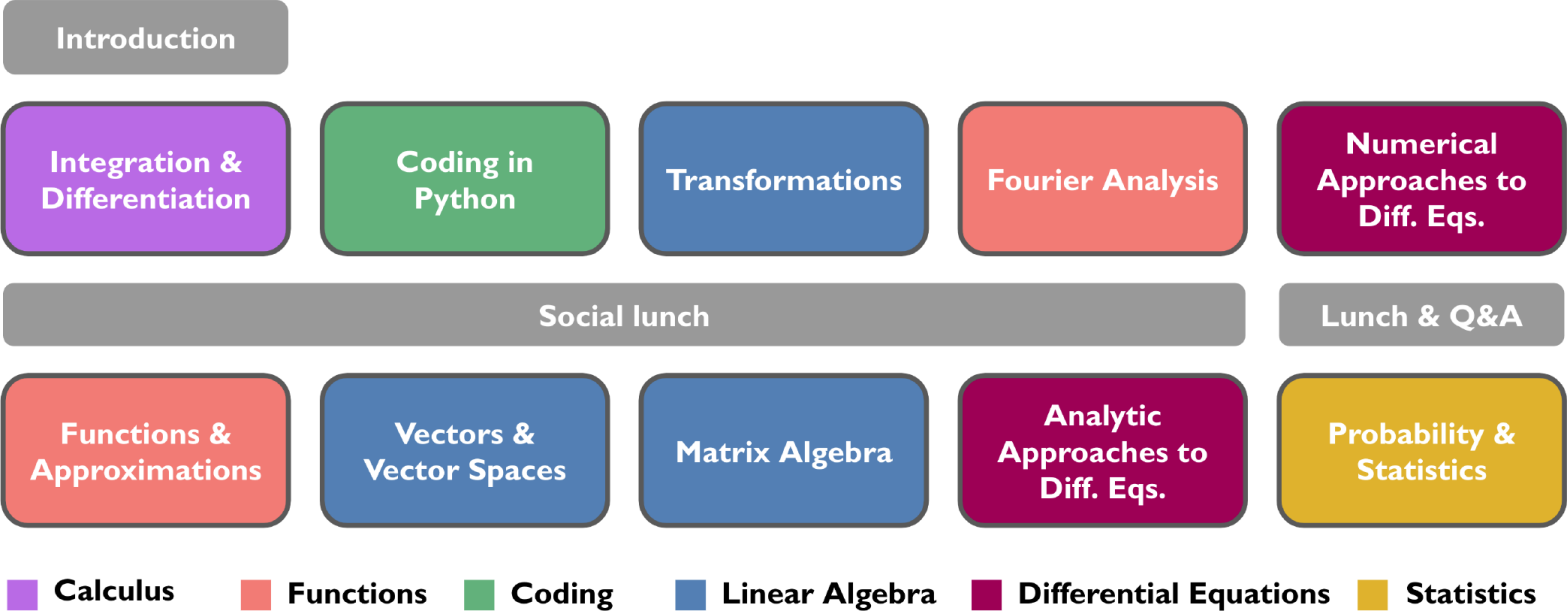
Schedule of the modules in the math bootcamp. Topics were chosen based on their relevance to introductory graduate coursework in physical chemistry.

Graduate coursework in physical chemistry requires a broad background in mathematics, and covering all relevant material in a week-long intervention like the bootcamp was therefore impossible. Instead, the scope of the content was narrowed to branches and subtopics in mathematics deemed to be most relevant for understanding lectures and completing problem sets in these introductory courses, which were identified through discussions with the professors teaching these courses and graduate students who had already taken them. Specifically, the bootcamp included modules on (1) single- and multivariable calculus; (2) functional analysis and approximation through methods like Taylor and Fourier series and expansions, finite differences, and plotting; (3) linear algebra with an emphasis on abstract vectors and vector spaces, transformations, and matrix algebra; (4) analytical and numerical approaches to the solution of differential equations; (5) probability and statistics; and (6) coding in Python. Where applicable, the curriculum design emphasized connections between these branches of mathematics. These topics were divided across a series of ten modules, as indicated in Figure 1.

A month prior to the start of the bootcamp, students received a PDF of bootcamp notes on each of these ten modules, complete with brief descriptions of the physical relevance of the topic, key definitions, summaries of concepts, and example problems with detailed written solutions. In order to accommodate diverse learning style preferences from nonmajoritarian cultures^47^ and to provide additional background, recommendations for publicly available, third-party resources in the form of links to educational videos or other sources of online notes were also provided to address both conceptual and computational aspects of each topic.^48,49^ This bootcamp packet, complete with links to these external references, is freely available under a Creative Commons BY-NC 4.0 license on the bootcamp website.^50^ Students were encouraged to work through these materials prior to the start of the bootcamp, which takes place the week before the new student orientation.

The ten bootcamp modules consist of 90 minute sessions spread out over the course of 5 days (Figure 1). A flipped-classroom model,^28^ where students are expected to engage with the provided material in advance of each session, was employed. While a brief (15–20 minute) lecture for each topic was delivered in order to establish common language, especially for students who may not have reviewed material beforehand, this structure dedicates the majority of the formal bootcamp sessions to solving new problems with the relevant material. Students are assigned to working groups of 3–4 people and work under the supervision of bootcamp instructors, who provide real-time feedback and help to ensure that each student is engaged. In 2020, this led to an instructor:student ratio of 1:5, while in 2021, the ratio was closer to 1:3. The instructors are upper-year graduate students who have completed at least one year of the chemistry Ph.D. program at UC Berkeley. The instructors are all physical chemists, although there is a mixture of experimental and theoretical chemists within that discipline. They were selected based on previous teaching experience and were interviewed by the founders of the bootcamp to ensure their teaching philosophies aligned with the goals of the project and reflect the diversity of the student body in gender, race, nationality, program year, and research background. This approach was taken on the basis of previous work demonstrating the effectiveness of peer-led team learning,^34,39^ process-oriented guided inquiry learning,^25^ and the role of a diverse instructor body in minimizing racially biased deficit theorization in the classroom.^51^ At the end of each session, all participants and instructors regroup to address common difficulties or overarching questions that students may have. Finally, PDFs of detailed solutions to the problems are posted at the end of each day.

The choices to include a variety of resource formats,^47^ center peer-led group problem-solving as outlined in the Introduction,^32−34^ and establish common language^2,14^ are aligned with critical frameworks and facilitate empowerment of all students. Throughout the bootcamp, significant care is taken to improve student confidence such that students feel safe bringing their whole selves to the discussion (i.e., feel free to ask for help, take risks, make mistakes), encouraging a cohesive social atmosphere.^2,14,52^ Specifically, at the beginning of the first day of bootcamp instruction, incoming students are introduced to the instructors and the bootcamp, emphasizing that the program is entirely student-run and that participant performance will not be conveyed to faculty. In this introduction, students are reassured that gaps in knowledge are normal and do not preclude their participation in the graduate program. Similarly, in both written and presented materials, care is taken to avoid language that presumes familiarity with topics. The bootcamp structure integrates regular and informal social times into the curriculum, allowing participants to get to know each other and the bootcamp instructors outside of the learning environment; these include a breakfast before the programming on the first day and lunches between the morning and afternoon modules throughout the bootcamp.

All aspects of the bootcamp—its inception, creation of content, instruction, facilitation of problem-solving groups, and follow-up procedures—were invented and spearheaded by graduate students in the Department of Chemistry at UC Berkeley. Following the successes of peer-led team learning in improving students’ attitudes,^32−34^ the authors believe that this “by students, for students” approach is a strong advantage of the program, further facilitating a sense of student belonging in bootcamp participants and easing the transition to graduate school.

## Methods

The impact of the bootcamp on students was evaluated by gathering data through surveys and interviews. The choice of research methods, data analysis techniques, and interpretations of results agree with general guidelines from a critical theory perspective for education research.^22,23^ Instead of focusing on quantitative data, such as individual student grades assigned by course instructors, the evaluatory surveys are centered on students’ responses to Likert-style questions and students’ experiences obtained as quotes.^53^ Additionally, quantitative data have been analyzed and interpreted to account for multiple intersections of identities among students, notwithstanding small sample sizes.^21^

Students who participated in the bootcamp had the option to complete three surveys, which are included in the Supporting Information (SI). The goal of the surveys was to evaluate the impact of the bootcamp on first-semester students in three distinct categories: (1) improving skills in mathematical computations related to the physical chemistry graduate curriculum, (2) increasing student confidence in important math topics, and (3) fostering a greater sense of belonging in the program and department. These surveys were reviewed by teaching faculty at UC Berkeley and participants in the College of Chemistry Graduate Diversity Program. Additionally, comments from students were considered after they participated in the bootcamp and completed the surveys each year.

The first, prebootcamp survey was given approximately one month before the bootcamp and gathered information about students’ confidence and mathematical preparedness for graduate coursework, and it included several short mathematical problems. The math problems for each student were selected randomly from a bank of questions (see Sections S2 and S5) such that the probability of any student solving the same problem in both the pre- and postbootcamp surveys would be low. This ensures that the students actually solve the problem and do not recall the answers from memory. The second, postbootcamp survey was administered shortly after the bootcamp ended and included math problems and asked about students’ confidence, similar to the presurvey, as well as which sessions they attended and which resources they used. The third, final survey was distributed at the end of the first semester and asked students if they continued using the bootcamp resources and assessed further changes in their confidence levels throughout the semester. In December 2021, all students received a consent form and a brief demographic questionnaire. This timeline corresponded to the end of the 2021 cohort’s first semester and the end of the 2020 cohort’s third semester. The demographic data that were collected pertained to the student’s gender identity, if they were a first-generation college/graduate student, if they were considered an international student, and whether they were part of an underrepresented minority in the field of chemistry.

Though the 2020 cohort included incoming first-year chemistry students, and the 2021 cohort included incoming first-year chemistry and chemical and biomolecular engineering students, the quantitative analysis below only includes physical chemistry students, as the bootcamp was tailored toward the first-semester physical chemistry courses. For the 2020 and 2021 bootcamp cohorts, 31 physical chemistry students responded to all three surveys and provided consent out of 67 total physical chemistry students participating in the bootcamp. Only those 31 students are included in this study, representing a response rate of 46.3%. Twenty-one of those students participated in the bootcamp in 2020 and 10 in 2021.

Students were surveyed on two distinct skill sets: math skills and academic social skills. Math skills are defined as skills related to the specific topics taught during the bootcamp, e.g., finding the eigenvalues and eigenvectors of a matrix. Academic social skills, by contrast, include things such as problem-solving as part of a group, confidence in asking professors or other instructors for help, and identifying external resources. Self-reported data on both academic and social confidence were collected on a scale of 1 to 5, with 5 being the highest confidence. Student responses were analyzed across topics and demographic groups and are described in more detail in the next section. Standard *t*-tests with individual *p*-values of 0.05 and added Bonferroni corrections^54,55^ were used to correct for the use of the same data set for testing multiple distinct hypotheses. The new *p*-value thresholds created by this correction are given in the next section. To describe the impact of the relationship between the different groups being compared by the *t*-tests, standardized mean differences are calculated using pooled standard deviations (Cohen’s *d*-value).^56^

The student text-responses to the open-ended survey questions were split into three general categories during analysis: sense of belonging, the transition to graduate education, and the usefulness of provided resources. Of the 31 students included in this study, 20 responded to the open-ended survey questions. Within these responses, 13 students mentioned sense of belonging, 13 students mentioned the transition to graduate education, and 3 students mentioned the usefulness of the provided resources. Many of the same students wrote comments about their sense of belonging and transition to graduate school, but the group that wrote about the usefulness of the bootcamp was disparate from those of either of the other categories. Throughout the analysis section, representative example quotes from these responses, which show the full range of the quantitative results being discussed, have been selected.

Data obtained from student surveys are supplemented by interviews with professors and graduate student instructors (GSIs) who taught the first-semester physical chemistry courses in 2020 and 2021. As the same two professors taught the courses both years, this amounted to interviewing six people, two professors, and four GSIs. A full set of interview questions may be found in Sections S8–S11. Interview data were analyzed for patterns in instructors’ observations regarding changes in students’ working in groups, students’ willingness to ask questions during class, and types of questions asked during office hours.

This research protocol was approved by the Institutional Review Board at UC Berkeley (ID: 2020–08–13583).

### Evaluating the Impact of the Bootcamp on Students

**Figure 2:**
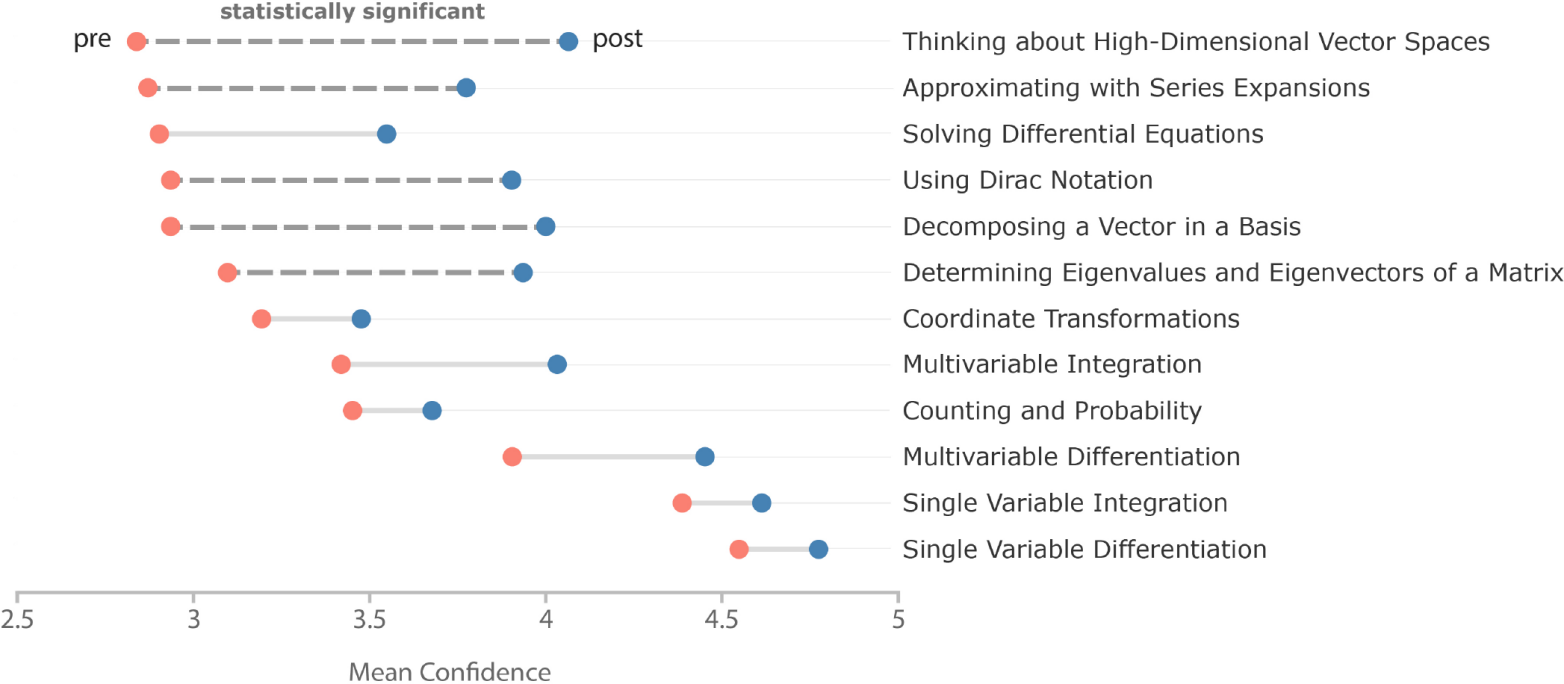
Mean student confidence in each math skill before and after the math bootcamp. Statistically significant changes, as evaluated in the Methods, are indicated by dashed lines in the dumbbell plot.

**Figure 3:**
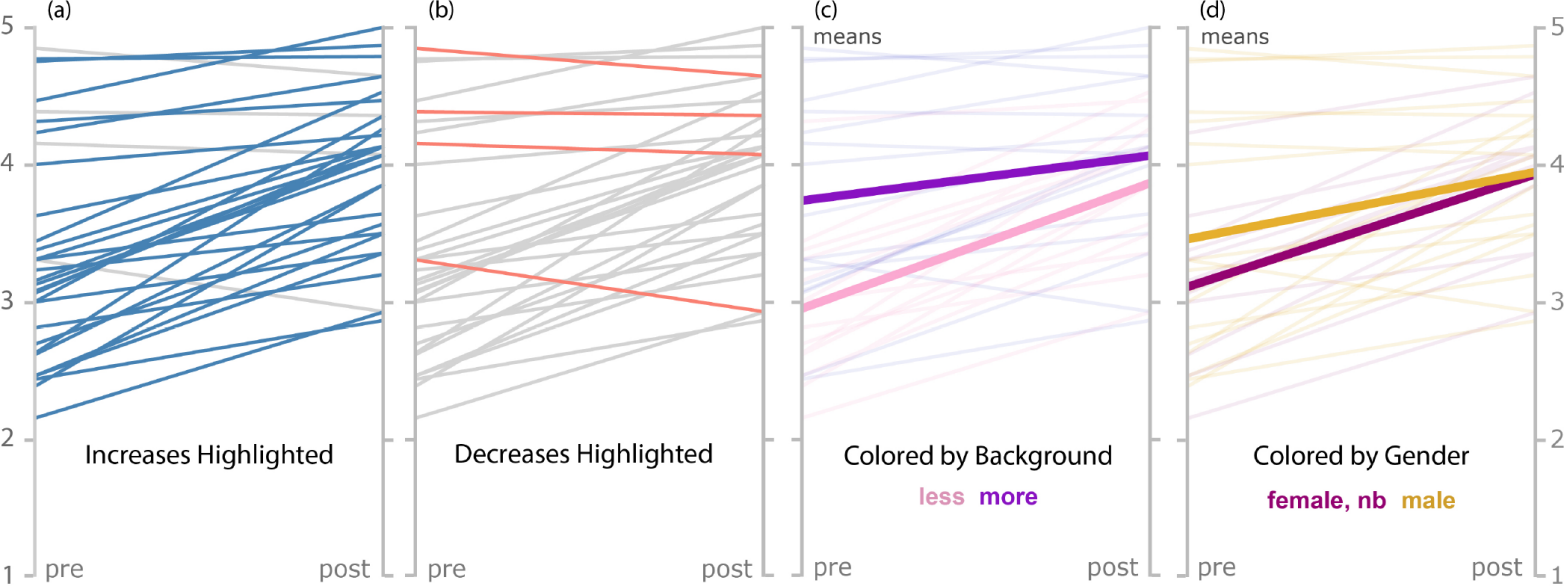
Mean confidence across math skills for each student. All figures include all students with different groups highlighted. Thick lines indicate the group averages. (a) Students whose confidence increased are highlighted in blue (*n* = 27). (b) Students whose confidence decreased are highlighted in orange (*n* = 4). (c) Students with more extensive math background—having taken linear algebra, differential equations, and multivariable calculus—are in purple (*n* = 17), and students with a less extensive math background—having not taken at least one of the aforementioned courses—are in pink (*n* = 14). (d) Male-identifying students are in yellow (*n* = 18), and female-identifying and nonbinary students are in mauve (*n* = 12).

Participation in the bootcamp increased mean self-reported student confidence across all studied skills, sometimes significantly, as illustrated in Figure 2. Data in Figure 3(a) show that the average increase in confidence comes from confidence gains from the vast majority of students, not from a few students having disproportionately large gains. Specifically, 27 students reported increased confidence while only 4 reported decreased confidence. Three of the four students who reported these decreases entered the bootcamp with higher than average self-reported confidence levels that fell only marginally after participation in the bootcamp (Figure 3(b)). Additionally, each of these four students participated in the bootcamp in 2020, which was hosted entirely virtually due to the COVID-19 pandemic. In 2021, when the bootcamp was offered in a hybrid format, the majority of students participated in-person, and all participants who contributed survey data reported confidence increases. The number of students who joined virtually varied day-to-day; however, typically only 5–7 of the 30 students joined via Zoom on any given day. Qualitative student responses further highlight difficulties with the virtual bootcamp format. For example, Student #1, a synthetic chemistry student whose confidence decreased from 3.8 to 3.7, reported that students did not engage very well during the breakout sessions, and even having an instructor drop in to help did not actually help.

This response indicates a breakdown in the active learning models^28,29^ in the virtual bootcamp format that negatively impacted this student’s experience. Stated differently, 5 students noted the importance of small group work in increasing their skills and confidence, and their experience suggests that in-person interactions can be more effective for learning than virtual ones. For example, Student #4 whose confidence increased from 3.4 to 4.5, reported:

I think the small groups worked well, at least in my experience. But I was also in-person, which I think was an advantage in that regard.

Table 1 reports the average change in scores for the five categories of math questions asked on the pre- and postbootcamp surveys. These results indicate that there was no measurable change in the aggregate student math ability. A lack of change in the score on average could arise from the students’ disparate levels of familiarity with the mathematical topics. Though the instructors focused on problem-solving in the bootcamp, some students may not have previously encountered the relevant algorithms that could be used to perform relevant calculations. The survey data from the mathematical questions were also difficult to analyze for trends because the students were randomly given one out of five different question options, which varied by specific topic and difficulty, in each of the mathematical categories.

**Table 1:** Average Change in Scores for Mathematics Problems in Pre- and Post-Surveys

Question	Avg.	Std. Error
Statistics	–0.07	0.12
Calculus	0.10	0.10
Differential Equations	0.14	0.11
Functions	0.00	0.12
Linear Algebra	–0.07	0.12

Still, students’ narrative responses to both surveys and course instructor interviews demonstrated that the bootcamp positively impacted students in their first-semester graduate coursework. Instructors of those courses noted that an increased number of students were familiar with more advanced mathematical topics—specifically Fourier transforms, probability, and linear algebra—and, furthermore, that students utilized outside resources to advance their knowledge of these topics much sooner in the semester. For example, the data collected in final surveys show that over a third of the respondents referred back to the provided bootcamp resources during the following semester.

While changes in problem-solving ability were not measurable, students’ *confidence* in their mathematical abilities increased significantly throughout the bootcamp, as shown in Figure 2. This increase was further noted by course instructors, who said that students’ questions at office hours pertained less to mathematical issues and instead focused on physical concepts compared to previous years. Student comments reflecting on the bootcamp also indicated that the bootcamp enabled them to make connections between mathematical concepts that they were unable to make in their undergraduate education. Student #5, whose self-reported confidence value increased by 1.06 during the bootcamp, writes:

I found math frustrating in college because I had trouble bridging the gap between coursework in math classes and its application to chemistry. This bootcamp helped me SO MUCH to finally make those connections that I’ve been needing for years and help me be able to use math (and coding) productively in my classes last fall.

Examination of aggregate confidence data reveals that larger average confidence increases were seen for students who lacked confidence upon entering the program and those from minoritized backgrounds. Specifically, students who entered the program with fewer completed courses in mathematics and nonmale-identifying students experienced larger confidence increases than their peers. Figure 3(d) compares confidence changes for students identifying as male versus students identifying as female or nonbinary. These data clearly show that underrepresented genders in mathematical fields had larger increases in confidence as a result of participating in the bootcamp than their male-identifying peers. Numerically, female and nonbinary students showed an increase of 0.82 ± 0.08 on average, while male students increased by 0.54 ± 0.07. While aggregate confidence increased for all students regardless of gender, the larger increase for nonmale students completely eliminated the gender gap that existed prior to the start of the bootcamp and that reflects gender gaps in math self-efficacy documented in the literature.^57,58^

Further analysis of the mathematical backgrounds of the students revealed that out of the 16 students entering graduate school having taken linear algebra, differential equations, and multivariable calculus, 11 were male-identifying (one student did not specify a gender identity). In contrast, the students who had not taken at least one of these courses upon entering graduate school were evenly split between male- and female/nonbinary-identifying students. This information shows that the students with underrepresented gender identities in this bootcamp had a less extensive mathematical background. Interestingly, the largest average confidence increases in Figure 3(c) were from male-identifying students with less extensive math backgrounds. This analysis, which is similar to those used in QuantCrit frameworks in critical theory for examining the simultaneous roles of race, gender, and competence in teacher evaluations,^59,60^ demonstrates that the gender inequity of mathematics confidence is independent from inequities in mathematics backgrounds, and that the bootcamp positively impacts both of these intersecting populations.

Data concerning the effect of the bootcamp on student confidence as a function of the students’ prior coursework experience are presented in Table 2. Specifically, changes in confidence levels between cohorts of students who did or did not take a given class in their undergraduate studies are compared. It is important to note that linear algebra, single variable calculus, multivariable calculus, and quantum mechanics had a relatively small number of students who reported not taking the course. A *t*-test analysis with Bonferroni corrections indicates a statistically significant difference in confidence gains between students who did and did not take single and multivariable calculus, differential equations, and linear algebra prior to the bootcamp. The measured effect sizes showed similar insight to the *p*-values from the *t*-test: the smallest *p*-values corresponded to larger effect sizes. For these four courses, students who had not taken the course reported much larger confidence gains than those who had taken these mathematical courses previously. It is interesting that prior experience with statistical and quantum mechanics did not result in meaningful differences for students. Prior exposure to physical chemistry did not influence measured confidence in mathematical ability despite close links between the topics. These results once again show that gaps between students’ confidence levels are shrinking as a result of the bootcamp. While an increase in confidence without a simultaneous increase in problem-solving ability may not reflect improvement in mathematical skills in the short term, a change in students’ self-reported efficacy has been shown to be positively correlated with long-term retention in mathematics and science education in high school and college and with attitude toward mathematics.^11,61−63^ It is expected that the increase in self-confidence will enable students to engage more with mathematics resources as they go through their graduate coursework.

**Table 2:** Average Change in Self-Reported Student Confidence^a^

	Did Take		Did Not Take			
Course Name	#	Average	#	Average	*p*-value	Effect Size
Differential Equations	19	0.39	12	1.04	0.00	0.71
Linear Algebra	24	0.55	7	0.99	0.00	0.47
Multivariable calculus	27	0.56	4	1.19	0.00	0.66
Single variable calculus	29	0.61	2	1.17	0.01	0.58
Statistics	11	0.55	20	0.70	0.14	0.16
Stat Mech	23	0.59	8	0.80	0.06	0.22
Quantum	28	0.64	3	0.67	0.89	0.02

a Average change in self-reported student confidence was based on the math courses students took during their undergraduate studies. The first four rows each have a *p*-value below a Bonferroni-corrected significance threshold of 0.71%.

Finally, in contrast to the course-by-course analysis in the preceding paragraph, this work considered differences in the effects of the bootcamp on students’ confidence for specific mathematical manipulations on the basis of the *total* undergraduate mathematics preparation of students (Table 3). Herein, students with “more” mathematical background are defined to have previously taken multivariable calculus, differential equations, and linear algebra. These courses were chosen as the comparison discussed in the previous paragraph showed that prior knowledge of these subjects had a noticeable impact on students’ confidence levels. A student having “less” mathematical background did not take at least one of those courses before starting the bootcamp. Most of the skill categories showed larger increases in confidence for the students labeled as having “less” background. Once again, *t*-tests were used to ensure differences between these two cohorts were statistically significant for each of the topics listed in the leftmost column of the table, and the resulting *p*-values are reported in the rightmost column of Table 3. While the changes for *individual* skills were not statistically significant on the basis of Bonferroni-corrected *p*-values, the difference between cohorts in *overall* change across all skills was significant and large, resulting in nearly 50% larger confidence increases for students with less previous mathematics exposure. This same trend can be seen in Figure 3(c). Based on the reported effect size values, there were very significant differences in confidence gains between the students with more/less mathematical background for the skills of “approximating functions with series expansions” and “determining the eigenvalues and eigenvectors of a matrix.” All of the other skills except for “decomposing a vector in a basis”, “using Dirac notation”, and “coordinate transformations” produced medium effect size values, showing that there is still a meaningful difference between the confidence changes of the two groups of students for a majority of these skills studied.

**Table 3:** Average Change in Student Self-Reported Confidence Levels Grouped Based on Whether Students Had “More” or “Less” of a Math Background Coming into Their First Year of Graduate School^a^

Math Skill	More Background	Less Background	*p*-Value	Effect Size
Approximating functions with series expansions	0.47	1.43	0.03	0.82
Solving differential equations	0.41	0.93	0.09	0.64
Decomposing a vector in a basis	0.82	1.36	0.22	0.46
Determining the eigenvalues and eigenvectors of a matrix	0.53	1.21	0.01	0.96
Thinking about high-dimensional vector spaces	0.88	1.64	0.04	0.76
Using Dirac notation	0.82	1.14	0.39	0.31
Coordinate transformations	0.18	0.57	0.20	0.48
Counting and probability	–0.06	0.57	0.09	0.64
Multivariable integration	0.41	0.86	0.11	0.60
Multivariable differentiation	0.24	0.71	0.10	0.61
Single variable integration	0.06	0.43	0.10	0.62
Single variable differentiation	0.06	0.43	0.07	0.69
Overall	0.45	0.87	0.00	0.46

a We defined “more” mathematical background as having previously taken multivariable calculus, linear algebra, and differential equations. If a student had not taken any one of those courses, they were put in the “less background” category. This led to 17 students being categorized as having more mathematical background and 14 students with less mathematical background. Using the Bonferroni correction required that the *p*-value for an individual category needed to fall below 0.42% to be considered statistically significant.

In comparison to the data for mathematics skills, quantitative changes in student confidence in social academic skills were less marked. Unlike mathematical confidence levels, which showed an increase in average confidence from 0.65 ± 0.05, social confidence increased by only 0.22 ± 0.10 as a result of the bootcamp. However, qualitative feedback from students and instructors showed a high degree of appreciation for the community-building that the bootcamp enabled before the start of the school year. Instructors anecdotally noted that students started working in groups on the homework and in office hours much more quickly than they had in previous years. Ten students commented on the relationships they were able to form with their peers during the bootcamp. For instance, Student #2, whose average confidence increased from 2.8 to 3.2, said:

After meeting in person and attending classes together, we worked on problem sets throughout the semester, which greatly reduced the stress of transitioning into grad school, gave reference for the workload of classes, and also helped foster a sense of belonging. Overall, I think the math bootcamp did an excellent job in increasing cohort camaraderie, giving a good overview for what to expect in terms of math theory/skills during coursework, and fostering relationships which will last through the [Ph.D.] program.

Likewise, Student #3, whose average confidence increased by 1.0, stated:

Honestly, I think the biggest thing it did was help me get to know some of the other students before classes started, and helped me see that we ALL were having a hard time, which I think helped me feel less bad about asking for help, etc. It also showed me we all had different strengths and I think really is responsible for my decision to work with people on the [problem sets], which is something I never did before.

These students’ comments support the notion that the bootcamp helped participants build valuable connections with other students in the program.

## Limitations

The qualitative and quantitative assessment of the impact of the bootcamp on graduate student learning experiences may have been skewed by several important limitations associated with the methods for data collection. Statistical analyses of all of the survey responses need to be interpreted in light of the small sample size of 31, consisting of the incoming physical chemistry graduate students over only two years at UC Berkeley who participated in the bootcamp and consented to having their response data analyzed. Specifically, since *t*-tests were used throughout the analysis to compare differences between different groups of students, this work assumed that, even with the limited amount of data, the data was approximately normally distributed in these comparisons and that there would be a similar amount of variance between the groups. The absence of data from a control group of students who did not participate in the bootcamp makes it difficult to interpret the magnitude of the changes in confidence. Additionally, the survey questions that tested the change in actual mathematical skills were optional and online, and it is possible that students were not sufficiently motivated to engage meaningfully with the questions. In the analysis, the authors assume that the students who filled out the survey still present an approximate representation of how the bootcamp impacted all students who participated. However, in the future, these limitations could be overcome by collecting data through additional years of operation of the bootcamp and by incorporating the survey administration into the main bootcamp schedule. Gathering survey data from a control group who does not benefit from participating in the bootcamp could be incentivized by compensating survey participants for their time.

The observed lack of change in the problem-solving score could also be potentially addressed by a longer-term intervention than the week-long bootcamp. Incoming graduate students have diverse backgrounds and hence could be impacted differently in the short- and long-term by a peer-led problem-solving practice. Recent work in the cognitive sciences has shown that general chemistry students solve chemistry problems by recalling facts and algorithms from long-term memory and applying them to the problem at hand.^64^ If students have not previously learned these algorithms, problem-solving sessions may not have an immediate positive impact. Further, as the bootcamp is scheduled immediately upon students’ first arrival at the university, it is not possible to tune the bootcamp content specifically to individual subgroups of students by gathering more information on their degree of automaticity in retrieval from long-term memory.

With regard to the data gathered from interviewing the instructors of the graduate physical chemistry courses, their perceptions of improvement in student performance might have been biased from their foreknowledge of the implementation of the bootcamp. This limitation can be partially mitigated by quantitatively analyzing aggregate student performance in exams and comparing to years before the bootcamp was developed, in the absence of confounding factors like the switch to remote learning.

## Future Work

In future iterations of the bootcamp, periodic review sessions on the bootcamp content throughout the first semester may strengthen the impact on mathematics competence. The bootcamp approach is most appropriate for students who have learned the bootcamp topics in previous courses.^64^ While students without this prerequisite knowledge may be best served by enrolling in the appropriate math coursework before enrolling in graduate physical chemistry coursework, these review sessions may facilitate the learning curve for all students.

Furthermore, the effect of increased mathematics confidence on students’ learning trajectories could also be directly monitored by measuring the competence at the end of the first semester through surveys or grades in physical chemistry courses. Attributing changes in competence from such a study would necessitate a control group who would not participate in the bootcamp but would be enrolled in the required graduate courses. A multiyear controlled study on the impact of the bootcamp that can account for the effects of prior mathematics background with a larger sample size would be useful to identify effective pedagogical approaches for a mathematics intervention for chemistry graduate students.

## Conclusion

The authors developed and conducted a peer-led mathematics bootcamp for incoming physical chemistry doctoral students at UC Berkeley. The bootcamp uses a flipped classroom approach to review and practice problem-solving pertaining to important topics in undergraduate mathematics over the course of 5 days. Self-reported surveys before and after the bootcamp indicated that the bootcamp increased confidence in the technical aspects of mathematical problem-solving and in solving problems in groups of peers. Comments from students and anecdotal evidence from professors support these findings and show that the bootcamp helped jumpstart community building within the cohort. Statistical analysis of these data demonstrates that the bootcamp significantly reduced inequities in mathematical confidence associated with prior mathematical background and gender identity.

In addition to these results, this work highlights recommendations for students at other universities who would like to implement such a bootcamp model for their own peers. The community building and increased confidence supported by the program are both expected to be important for helping students thrive in the physical sciences and thus should be supported by departments in the form of fair compensation to instructors from the department for the significant time and effort involved in fine-tuning content and executing the bootcamp. Cooperation from the department can be highly effective in establishing first contact with incoming students to inform them about the bootcamp. In the same spirit, any efforts from the department to encourage students to arrive a week before orientation events start, such as by offering temporary housing, seem likely to improve the level of bootcamp participation. These measures can help the instructors institutionalize the bootcamp for smooth functioning in subsequent years. As for the choice of new bootcamp instructors, those involved in previous years as well as the teaching assistants for the graduate physical chemistry courses are expected to have superior knowledge of the unique challenges faced by new students in terms of mathematical skills and hence should be recruited.

This bootcamp is not intended to replace the need for improved graduate curricula that adequately teaches physical chemistry students the key mathematical topics that are usually prerequisites for graduate courses. Employing a critical perspective, we find that enrollment in relevant mathematics courses is decreased among students that are historically minoritized in math, specifically those that identify as female or nonbinary. Hence, achieving equity in graduate physical chemistry education requires systemic, institutional changes that address mathematics pedagogy using a critical framework instead of focusing on adapting minoritized groups so that they fit educational systems designed originally for white male students.^14,17^ However, the present results provide encouragement to physical chemistry doctoral students in other universities to use similar mathematics bootcamps to improve the academic experience and start community-building among incoming students. Results in the preceding sections indicate that mathematics bootcamps, when intentionally designed to empower diverse cohorts, can contribute positively to equitable outcomes by giving all students the opportunity to start graduate school on a more equal footing, regardless of their background. Employing a flipped classroom approach and centering bootcamp time around group-work facilitate connections between new students, encourage equal participation, and normalize asking for help in graduate school. The community building opportunities the bootcamp provides improve students’ sense of belonging and ease the transition to graduate school.

## Supporting Information Available

Additional results and protocols for surveys and interviews (PDF)

## Related Paper

A preprint of this manuscript is available on ChemRxiv.^65^
