# Supplementary material for: Development of a Week-Long Mathematics Intervention for Incoming Chemistry Graduate Students
Source: J Chem Educ. 2023 Aug 24;100(9):3291–301. doi: 10.1021/acs.jchemed.2c00915 (PMC10501113; doi:10.1021/acs.jchemed.2c00915)
Supplement: Supplementary file 1 — ed2c00915_si_001.pdf [file ed2c00915_si_001.pdf]

Supporting Information: Development of a week-long mathematics intervention for incoming chemistry graduate students

Rachel Clune,[*,†,‡,¶] Avishek Das,[*,†,‡,¶,#] Dipti Jasrasaria,[*,†,¶,@] Elliot Rossomme,[*,§,‡,¶,§] Orion Cohen,[*,‖,‖,¶] and Anne M. Baranger[*,¶,⊥]

†*These authors contributed equally and are listed alphabetically by last name*

‡*Kenneth S. Pitzer Center for Theoretical Chemistry, University of California, Berkeley, CA 94720, United States*

¶*Department of Chemistry, University of California, Berkeley, CA 94720, United States*

§*Chemical Sciences Division, Lawrence Berkeley National Laboratory, Berkeley, California 94720, United States*

‖*Materials Science Division, Lawrence Berkeley National Laboratory, Berkeley, CA, 94720, United States*

⊥*Graduate Group in Science and Mathematics Education, University of California, Berkeley, CA 94720, United States*

#*Current address: AMOLF, Science Park 102, 1098 XG, Amsterdam, The Netherlands; a.das@amolf.nl*

@*Current address: Department of Chemistry, Columbia University, New York, New York 10027, United States; dj2667@columbia.edu*

E-mail: rclune4b@berkeley.edu; avishek_das@berkeley.edu; djasrasaria@berkeley.edu; elliot_rossomme@berkeley.edu; orioncohen@berkeley.edu; abaranger@berkeley.edu

Contents

**S1**	**Change in self-reported confidence**	**S3**
**S2**	**2020 Pre-Survey**	**S5**
**S3**	**2021 Pre-Survey**	**S19**
**S4**	**2020 Post-Survey**	**S33**
**S5**	**2021 Post-Survey**	**S47**
**S6**	**2020 Final Survey**	**S61**
**S7**	**2021 Final Survey**	**S67**
**S8**	**2020 Professor Interview Questions**	**S73**
**S9**	**2021 Professor Interview Questions**	**S75**
**S10**	**2020 GSI Interview Questions**	**S77**
**S11**	**2021 GSI Interview Questions**	**S79**

S1 Change in self-reported confidence

Table S1: Average change in self-reported confidence from pre and post surveys.

Question	Average	Standard error
Approximating functions with series expansions	0.90	0.22
Solving differential equations	0.65	0.15
Decomposing a vector in a basis	1.06	0.21
Determining the eigenvalues and eigenvectors of a matrix	0.84	0.14
Thinking about high-dimensional vector spaces	1.23	0.19
Using Dirac notation	0.97	0.18
Coordinate Transformations	0.35	0.15
Counting and probability	0.23	0.18
Multivariable integration	0.61	0.14
Multivariable differentiation	0.45	0.15
Single variable integration	0.23	0.11
Single variable differentiation	0.23	0.10
Solving problems in a group	0.48	0.17
Asking professors or TAs/GSIs about material	0.10	0.14
Identifying relevant online or textbook resources	0.06	0.20
Overall	0.56	0.05

S2 2020 Pre-Survey

Preamble

The purpose of this survey is to provide us with a better understanding of the math background of your incoming cohort and to sign up for the bootcamp. **We welcome responses from all of you even if you don't plan to participate in the bootcamp, because it will help us gauge its utility.**

The survey consists of brief questions about your familiarity and comfort with certain math concepts, 5 very short answer style math questions, and questions about which courses you have taken and are planning to take. **The survey may take you about 15 minutes to complete.**

The survey is not intended to be taken on mobile devices, which will not have optimized image formatting. If possible, we recommend completing it on a desktop or laptop computer.

General

Name

Email

Email:

Confirm Email:

Will you be participating in the Math Bootcamp (Aug 6-12)?

- Yes
- No

Confidence

Indicate your comfort level with each of the following branches of mathematics (1 = very uncomfortable, 5 = very comfortable):

	1	2	3	4	5
Single variable integration	○	○	○	○	○
Multivariable integration	○	○	○	○	○
Single variable differentiation	○	○	○	○	○
Multivariable differentiation	○	○	○	○	○
Coordinate transformations (polar, spherical, Cartesian, etc.)	○	○	○	○	○
Solving differential equations	○	○	○	○	○
Approximating functions with series expansions	○	○	○	○	○
Decomposing a vector in a basis	○	○	○	○	○
Determining the eigenvalues and eigenvectors of a matrix	○	○	○	○	○
Using Dirac notation	○	○	○	○	○
Thinking about high-dimensional vector spaces	○	○	○	○	○
Counting and probability	○	○	○	○	○

Indicate your comfort level with each of the following activities as pertaining to math-related content (1 = very uncomfortable, 5 = very comfortable):

	1	2	3	4	5
Solving problems in a group	○	○	○	○	○
Asking professors or TAs/GSIs about material	○	○	○	○	○

	1	2	3	4	5
Identifying relevant online or textbook resources	○	○	○	○	○

Math preamble

The following 5 short answer style math questions will help us gauge your familiarity with certain types of math concepts and questions, and will help us determine the utility and effectiveness of the math bootcamp.

For each question, please write the short answer or the letter of the best provided answer. You may want to use some scratch paper to help you work out your answer. Please do your best to answer each question.

It is totally okay if you are unable to answer all (or any!) of the following problems. We want to emphasize that solving arbitrary math problems with minimal context and warmup does not reflect your potential for being a successful student or researcher. **Regardless of what our imposter syndrome may tell us, all of us belong here, in this department.** The sole purpose of these questions is to understand the effectiveness of the bootcamp in building skills and confidence to answer these specific types of math problems.

Probability

Is $f(r) = \frac{4}{a^3} r^2 \exp(-2r/a)$ a valid probability distribution?

(a) Yes

(b) Yes, but only for $r > 0$

(c) No, it's not normalized

(d) No, $f(r)$ cannot be greater than 1

Evaluate the following integral: $I = \int_{-\infty}^{\infty} \exp(-\frac{x^2}{2\sigma^2})$

(a) $I = 1$

(b) $I = 1/\sqrt{2\pi\sigma^2}$

(c) $I = \sqrt{2\pi\sigma^2}$

(d) $I = \pi$

How many ways can n indistinguishable particles be placed in k indistinguishable boxes?

(a) $\frac{n!}{k!}$

(b) $\binom{n}{k} = \frac{n!}{k!(n-k)!}$

(c) $\frac{n!}{(n-k)!}$

(d) $\binom{n+k-1}{k-1} = \binom{n+k-1}{n} = \frac{(n+k-1)!}{n!(k-1)!}$

Let $E(x)$ be a function describing the energy of a particle as a function of its position x. Which distribution would you use to evaluate the energy of a particle constrained to a certain position x_0?

(a) Gaussian

(b) Dirac delta

(c) Poisson

(d) Binomial

A random variable X has a mean of μ and variance of σ^2. Let another random variable $Y = 3X + 4$. What is the variance of Y?

How confident were you in your ability to answer this question?

- 1 Not at all confident
- 2
- 3 Somewhat confident
- 4
- 5 Very confident

Calculus

What is the shape of the region of xyz-space where $x+y+z \leq 1$ and $x, y, z \geq 0$?

(a) Cone

(b) Ellipsoid

(c) Paraboloid

(d) Rectangular Prism

(e) Tetrahedron

Suppose that $z = f(x(t), y(t))$ where $\left(\frac{\partial z}{\partial x}\right)_y = 3$, $\left(\frac{\partial z}{\partial y}\right)_x = 1$, $\frac{dx}{dt} = 7$, $\frac{dx}{dt} = 2$. What is $\frac{dz}{dt}$?

What is the slope of $f(x, y) = 2x^3y + y$ in the y-direction at $(1, -1)$?

In a Taylor Series, what does the constant term represent?

When changing variables from Cartesian to spherical coordinates the volume differential $dx\,dy\,dz$ is replaced by $r^2 \sin\phi\, dr\, d\phi\, d\theta$. Explain the factor of r^2 and the factor of $\sin\phi$.

How confident were you in your ability to answer this question?

- 1 Not at all confident
- 2
- 3 Somewhat confident
- 4
- 5 Very confident

Differential Equations

The ODE $\frac{d^2y}{dx^2} = 0$ represents $y(x)$ as a

(a) Family of horizontal lines

(b) Family of vertical lines

(c) Family of straight lines with a fixed slope

(d) Set of arbitrary straight lines

What is the dimension of phase space for a particle trajectory in 3D?

Which integrating factor will make the ODE $(x \ln x)dy + ydx = 0$ exact?

(a) x

(b) $1/x$

(c) $1/\ln x$

What is the general solution of $\frac{d^2y}{dx^2} - 3\frac{dy}{dx} + 2y = 0$?

The infinite power series that will solve the ODE $\frac{dy}{dx} = 1 - xy$ is

(a) $y = 1 + x + \frac{x^2}{2!} + \frac{x^3}{3!} + ...$

(b) $y = 1 + \frac{x^2}{(2)(1)} + \frac{x^4}{(4)(3)} + ...$

(c) $y = x - \frac{x^3}{(3)(1)} + \frac{x^5}{(5)(3)(1)} - ...$

(d) $y = x - \frac{x^3}{3} + \frac{x^5}{5} - ...$

How confident were you in your ability to answer this question?

- 1 Not at all confident
- 2
- 3 Somewhat confident
- 4
- 5 Very confident

Functions

What is the sum of $1 - \frac{1}{2} + \frac{1}{4} - \frac{1}{8} + ...$?

The Fourier series expansion of $y = 1/(x - x^5)$ will have

(a) Only cosines

(b) Only sines

(c) Cosine coefficients = corresponding sine coefficients

(d) Cosine coefficients = − corresponding sine coefficients

The power series expansion for $\sinh x = \frac{e^x - e^{-x}}{2}$ is

(a) $1 + \frac{x^2}{2!} + \frac{x^4}{4!} + ...$

(b) $x + \frac{x^3}{3!} + \frac{x^5}{5!} + ...$

(c) $x - \frac{x^3}{3!} + \frac{x^5}{5!} - ...$

What are the diagonal elements of the double derivative operator d^2/dx^2 in this exponential space $\{1, e^{-x}, e^{-2x}, e^{-3x}, ...\}$?

The Fourier transform $g(\omega)$ of a function $f(t)$ is to be defined as

$$g(\omega) = \frac{1}{\sqrt{2\pi}} \int_{-\infty}^{\infty} f(t) e^{i\omega t} dt$$

Match the functions to their corresponding Fourier transforms in the following two figures.

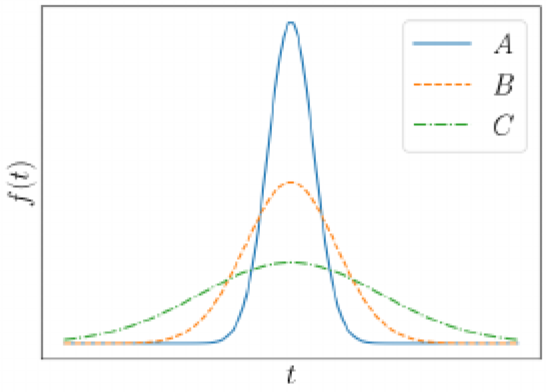

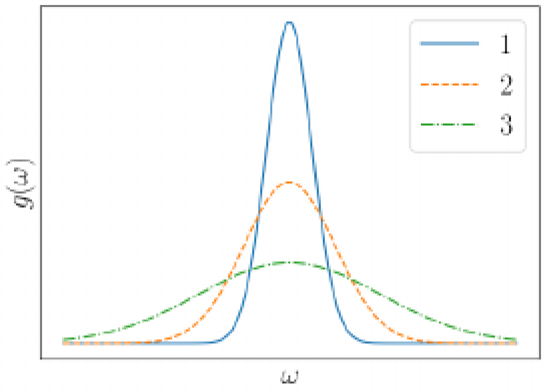

How confident were you in your ability to answer this question?

- 1 Not at all confident
- 2
- 3 Somewhat confident
- 4
- 5 Very confident

Linear Algebra

What is the dimension of the space spanned by the following two vectors?

$$\begin{bmatrix} 2 \\ 3 \\ 1 \end{bmatrix} \qquad \begin{bmatrix} 1 \\ 0 \\ -1 \end{bmatrix}$$

What are the eigenvalues of the matrix $\mathbf{A}$?

$$\mathbf{A} = \begin{bmatrix} 4 & 2 & 0 \\ 1 & 5 & 0 \\ 0 & 0 & -3 \end{bmatrix}$$

Which of the following vectors is orthogonal to the vector $\mathbf{v}$? (Select all that apply)

$$\mathbf{v} = \begin{bmatrix} 2 \\ -3 \\ i \end{bmatrix}$$

$$\mathbf{a} = \begin{bmatrix} i \\ 0 \\ -2 \end{bmatrix}, \quad \mathbf{b} = \begin{bmatrix} 1 \\ 1 \\ i \end{bmatrix}, \quad \mathbf{c} = \begin{bmatrix} 1+i \\ 0 \\ 2 \end{bmatrix}, \quad \mathbf{d} = \begin{bmatrix} 3 \\ -2 \\ 1 \end{bmatrix}$$

- ☐ a
- ☐ b
- ☐ c
- ☐ d

Expressed in the basis $|a\rangle, |b\rangle, |c\rangle, |d\rangle$, the operator $\mathbf{A}$ has the following matrix representation:

$$\begin{bmatrix} 1 & 0 & -3 & 0 \\ 0 & -1 & 0 & 0 \\ 2 & 0 & -2 & 0 \\ 0 & 0 & 0 & 4 \end{bmatrix}.$$

Which (if any) of $|a\rangle, |b\rangle, |c\rangle$, and $|d\rangle$ are eigenvectors of $\mathbf{A}$? Select all that apply. (You do not need to explicitly solve for the eigensystem of $\mathbf{A}$ in solving this problem.)

(a) $|a\rangle$

(b) $|b\rangle$

(c) $|c\rangle$

(d) $|d\rangle$

- ☐ a

- [ ] b
- [ ] c
- [ ] d

Which of the following are linear operators? (Select all that apply)

(a) $\hat{A} = x$

(b) $\hat{B} = \frac{\partial}{\partial x}$

(c) $\hat{C} = \int_{-\infty}^{\infty} dx$

(d) $\hat{D} = \frac{\partial}{\partial x} x$

- [ ] A
- [ ] B
- [ ] C
- [ ] D

How confident were you in your ability to answer this question?

- ○ 1 Not at all confident
- ○ 2
- ○ 3 Somewhat confident
- ○ 4
- ○ 5 Very confident

Preparedness

If you enrolled in the Chemistry PhD program, which tract are you in?

- ○ Physical chemistry
- ○ Synthetic chemistry
- ○ Chemical biology
- ○ Other (please specify) ________

Which graduate course(s) are you planning on taking this semester?

- ☐ Thermodynamics and Statistical Mechanics (CHEM 220A)
- ☐ Advanced Quantum Mechanics (CHEM 221A)
- ☐ Quantum Mechanics (PHYS 221A)
- ☐ I don't know yet
- ☐ Other (please specify) ____

Which undergraduate physical science courses have you taken? Select all that apply and write the number of semesters for each subject, using fractions if multiple subjects were combined in a one-semester course.

- ☐ Thermodynamics ____
- ☐ Statistical mechanics (in a chemistry department) ____
- ☐ Statistical mechanics (in a physics department) ____
- ☐ Quantum mechanics (in a chemistry department) ____
- ☐ Quantum mechanics (in a physics department) ____
- ☐ Physics - Classical mechanics ____
- ☐ Physics - Electricity and magnetism ____
- ☐ Other undergraduate coursework (please specify) ____
- ☐ Graduate coursework (please specify) ____

Which undergraduate mathematics courses have you taken? Select all that apply and write the number of semesters for each subject, using fractions if multiple subjects were combined in a one-semester course.

- ☐ Single variable calculus (typically Calculus I & II) ____
- ☐ Multivariable calculus (typically Calculus III) ____
- ☐ Linear algebra ____
- ☐ Differential equations ____
- ☐ Statistics ____
- ☐ Mathematical Methods of Physics ____
- ☐ Other undergraduate coursework (please specify) ____

☐ Graduate coursework (please specify) ____________

Powered by Qualtrics

S3 2021 Pre-Survey

Preamble

The purpose of this survey is to provide us with a better understanding of the math background of your incoming cohort and to sign up for the bootcamp**. We welcome responses from all of you even if you don't plan to participate in the bootcamp, because it will help us gauge its utility.**

The survey consists of brief questions about your familiarity and comfort with certain math concepts, 5 short-answer style math questions, and questions about which courses you have taken and are planning to take**. The survey may take you about 15 minutes to complete.**

The survey is not intended to be taken on mobile devices, which will not have optimized image formatting. If possible, we recommend completing it on a desktop or laptop computer.

General

Name

Email (@berkeley address, if possible)

Email:

Confirm Email:

Will you be participating in the Math Bootcamp (Aug 5-11)? We plan to offer all bootcamp sessions in person, but we will also have the option for students to join remotely.

- ○ Yes, I will attend in person
- ○ Yes, I will attend via Zoom

○ I'm not sure yet

○ No, I will not be participating

○ Other __________

Confidence

Indicate your comfort level with each of the following branches of mathematics (1 = very uncomfortable, 5 = very comfortable):

	1	2	3	4	5
Single variable integration	○	○	○	○	○
Multivariable integration	○	○	○	○	○
Single variable differentiation	○	○	○	○	○
Partial differentiation	○	○	○	○	○
Coordinate transformations (polar, spherical, Cartesian, etc.)	○	○	○	○	○
Solving differential equations	○	○	○	○	○
Approximating functions with series expansions	○	○	○	○	○
Fourier analysis	○	○	○	○	○
Plotting graphs on a computer	○	○	○	○	○
Decomposing a vector in a basis	○	○	○	○	○
Determining the eigenvalues and eigenvectors of a matrix	○	○	○	○	○
Using Dirac notation	○	○	○	○	○
Thinking about high-dimensional vector spaces	○	○	○	○	○
Counting and probability	○	○	○	○	○
Writing or reading computer code	○	○	○	○	○

Indicate your comfort level with each of the following activities as pertaining to math-related content (1 = very uncomfortable, 5 = very comfortable):

	1	2	3	4	5
Solving problems in a group	○	○	○	○	○
Asking professors or TAs/GSIs about material	○	○	○	○	○
Identifying relevant online or textbook resources	○	○	○	○	○
Learning / working over Zoom	○	○	○	○	○

Math preamble

The following 5 short-answer style math questions will help us gauge your familiarity with certain types of math concepts and questions, and will help us determine the utility and effectiveness of the math bootcamp.

For each question, please write the short answer or the letter of the best provided answer. You may want to use some scratch paper to help you work out your answer. Please do your best to answer each question.

It is totally okay if you are unable to answer all (or any!) of the following problems. We want to emphasize that solving arbitrary math problems with minimal context and warmup does not reflect your potential for being a successful student or researcher. **Regardless of what our imposter syndrome may tell us, all of us belong here, in this department.** The sole purpose of these questions is to understand the effectiveness of the bootcamp in building skills and confidence to answer these specific types of math problems.

Probability

Is $f(r) = \frac{4}{a^3} r^2 \exp(-2r/a)$ a valid probability distribution?

(a) Yes

(b) Yes, but only for $r > 0$

(c) No, it's not normalized

(d) No, $f(r)$ cannot be greater than 1

Evaluate the following integral: $I = \int_{-\infty}^{\infty} \exp(-\frac{x^2}{2\sigma^2})$

(a) $I = 1$

(b) $I = 1/\sqrt{2\pi\sigma^2}$

(c) $I = \sqrt{2\pi\sigma^2}$

(d) $I = \pi$

How many ways can n indistinguishable particles be placed in k indistinguishable boxes?

(a) $\frac{n!}{k!}$

(b) $\binom{n}{k} = \frac{n!}{k!(n-k)!}$

(c) $\frac{n!}{(n-k)!}$

(d) $\binom{n+k-1}{k-1} = \binom{n+k-1}{n} = \frac{(n+k-1)!}{n!(k-1)!}$

Let $E(x)$ be a function describing the energy of a particle as a function of its position x. Which distribution would you use to evaluate the energy of a particle constrained to a certain position x_0?

(a) Gaussian

(b) Dirac delta

(c) Poisson

(d) Binomial

A random variable X has a mean of μ and variance of σ^2. Let another random variable $Y = 3X + 4$. What is the variance of Y?

How confident were you in your ability to answer this question?

- 1 Not at all confident
- 2
- 3 Somewhat confident
- 4
- 5 Very confident

Calculus

What is the shape of the region of xyz-space where $x+y+z \leq 1$ and $x, y, z \geq 0$?

(a) Cone

(b) Ellipsoid

(c) Paraboloid

(d) Rectangular Prism

(e) Tetrahedron

Suppose that $z = f\big(x(t), y(t)\big)$ where $\left(\frac{\partial z}{\partial x}\right)_y = 3$, $\left(\frac{\partial z}{\partial y}\right)_x = 1$, $\frac{dy}{dt} = 7$, $\frac{dx}{dt} = 2$. What is $\frac{dz}{dt}$?

What is the slope of $f(x, y) = 2x^3y + y$ in the y-direction at $(1, -1)$?

Where does the constant term in the Taylor expansion of a function come from?

When changing variables from Cartesian to spherical coordinates, the volume differential $dx\,dy\,dz$ is replaced by $r^2 \sin\phi\, dr\, d\phi\, d\theta$. Why do we include the factors of r^2 and $\sin\theta$?

How confident were you in your ability to answer this question?

- 1 Not at all confident
- 2
- 3 Somewhat confident
- 4
- 5 Very confident

Differential Equations

The ODE $\frac{d^2y}{dx^2} = 0$ represents $y(x)$ as a

(a) Family of horizontal lines

(b) Family of vertical lines

(c) Family of straight lines with a fixed slope

(d) Set of arbitrary straight lines

What is the dimension of phase space for a particle trajectory in 3D?

Which integrating factor will make the ODE $(x \ln x)dy + ydx = 0$ exact?

(a) x

(b) $1/x$

(c) $1/\ln x$

What is the general solution of $\frac{d^2y}{dx^2} - 3\frac{dy}{dx} + 2y = 0$?

The infinite power series that will solve the ODE $\frac{dy}{dx} = 1 - xy$ is

(a) $y = 1 + x + \frac{x^2}{2!} + \frac{x^3}{3!} + ...$

(b) $y = 1 + \frac{x^2}{(2)(1)} + \frac{x^4}{(4)(3)} + ...$

(c) $y = x - \frac{x^3}{(3)(1)} + \frac{x^5}{(5)(3)(1)} - ...$

(d) $y = x - \frac{x^3}{3} + \frac{x^5}{5} - ...$

How confident were you in your ability to answer this question?

- 1 Not at all confident
- 2
- 3 Somewhat confident
- 4
- 5 Very confident

Functions

What is the sum of $1 - \frac{1}{2} + \frac{1}{4} - \frac{1}{8} + ...$?

The Fourier series expansion of $y = 1/(x - x^5)$ will have

(a) Only cosines

(b) Only sines

(c) Cosine coefficients = corresponding sine coefficients

(d) Cosine coefficients = − corresponding sine coefficients

The power series expansion for $\sinh x = \frac{e^x - e^{-x}}{2}$ is

(a) $1 + \frac{x^2}{2!} + \frac{x^4}{4!} + \ldots$

(b) $x + \frac{x^3}{3!} + \frac{x^5}{5!} + \ldots$

(c) $x - \frac{x^3}{3!} + \frac{x^5}{5!} - \ldots$

What are the diagonal elements of the double derivative operator d^2/dx^2 in the space of imaginary exponentials $\{1, e^{ix}, e^{2ix}, e^{3ix}, \ldots\}$?

The Fourier transform $g(\omega)$ of a function $f(t)$ is to be defined as

$$g(\omega) = \frac{1}{\sqrt{2\pi}} \int_{-\infty}^{\infty} f(t) e^{i\omega t} dt$$

Match the functions to their corresponding Fourier transforms in the following two figures.

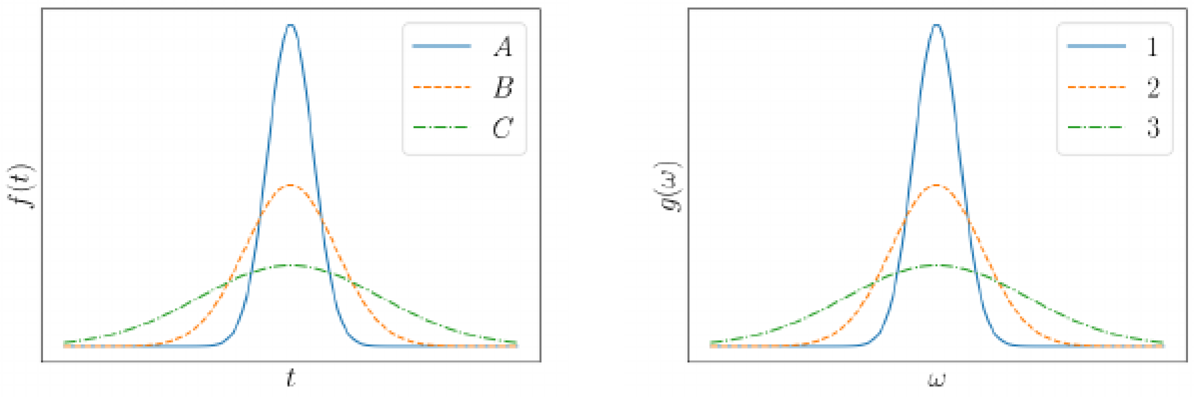

How confident were you in your ability to answer this question?

- 1 Not at all confident
- 2
- 3 Somewhat confident
- 4
- 5 Very confident

Linear Algebra

What is the dimension of the space spanned by the following two vectors?

$$\begin{bmatrix} 2 \\ 3 \\ 1 \end{bmatrix} \quad \begin{bmatrix} 1 \\ 0 \\ -1 \end{bmatrix}$$

What are the eigenvalues of the matrix $\mathbf{A}$?

$$\mathbf{A} = \begin{bmatrix} 4 & 2 & 0 \\ 1 & 5 & 0 \\ 0 & 0 & -3 \end{bmatrix}$$

Which of the following vectors is orthogonal to the vector $\mathbf{v}$? (Select all that apply)

$$\mathbf{v} = \begin{bmatrix} 2 \\ -3 \\ i \end{bmatrix}$$

$$\mathbf{a} = \begin{bmatrix} i \\ 0 \\ -2 \end{bmatrix}, \qquad \mathbf{b} = \begin{bmatrix} 1 \\ 1 \\ i \end{bmatrix}, \qquad \mathbf{c} = \begin{bmatrix} 1+i \\ 0 \\ 2 \end{bmatrix}, \qquad \mathbf{d} = \begin{bmatrix} 3 \\ -2 \\ 1 \end{bmatrix}$$

- ☐ a
- ☐ b
- ☐ c
- ☐ d

Expressed in the basis $|a\rangle, |b\rangle, |c\rangle, |d\rangle$, the operator $\mathbf{A}$ has the following matrix representation:

$$\begin{bmatrix} 1 & 0 & -3 & 0 \\ 0 & -1 & 0 & 0 \\ 2 & 0 & -2 & 0 \\ 0 & 0 & 0 & 4 \end{bmatrix}.$$

Which (if any) of $|a\rangle, |b\rangle, |c\rangle$, and $|d\rangle$ are eigenvectors of $\mathbf{A}$? Select all that apply. (You do not need to explicitly solve for the eigensystem of $\mathbf{A}$ in solving this problem.)

(a) $|a\rangle$

(b) $|b\rangle$

(c) $|c\rangle$

(d) $|d\rangle$

- ☐ a

- ☐ b
- ☐ c
- ☐ d

Which of the following are linear operators? (Select all that apply)

(a) $\hat{A} = x$

(b) $\hat{B} = \frac{\partial}{\partial x}$

(c) $\hat{C} = \int_{-\infty}^{\infty} dx$

(d) $\hat{D} = \frac{\partial}{\partial x} x$

- ☐ A
- ☐ B
- ☐ C
- ☐ D

How confident were you in your ability to answer this question?

- ○ 1 Not at all confident
- ○ 2
- ○ 3 Somewhat confident
- ○ 4
- ○ 5 Very confident

Preparedness

If you enrolled a PhD program in the College of Chemistry, which program / track are you in?

- ○ Physical chemistry
- ○ Synthetic chemistry
- ○ Chemical biology
- ○ Chemical and biomolecular engineering
- ○ Other (please specify) ____________

Which graduate course(s) are you planning on taking this semester?

- [ ] Thermodynamics and Statistical Mechanics (CHEM 220A)
- [ ] Advanced Quantum Mechanics (CHEM 221A)
- [ ] Quantum Mechanics (PHYS 221A)
- [ ] I don't know yet
- [ ] Other (please specify) ____________

Which undergraduate physical science courses have you taken for a full semester / term?

- [ ] Thermodynamics
- [ ] Statistical mechanics (in a chemistry department)
- [ ] Statistical mechanics (in a physics department)
- [ ] Quantum mechanics (in a chemistry department)
- [ ] Quantum mechanics (in a physics department)
- [ ] Physical chemistry
- [ ] Physics - Classical mechanics
- [ ] Physics - Electricity and magnetism
- [ ] Other undergraduate coursework (please specify) ____________
- [ ] Graduate coursework (please specify) ____________

Which undergraduate mathematics courses have you taken for a full semester / term?

- [ ] Single variable calculus (typically Calculus I & II)
- [ ] Multivariable calculus (typically Calculus III)
- [ ] Linear algebra
- [ ] Differential equations
- [ ] Statistics / probability
- [ ] Math for the physical sciences
- [ ] Computer science / Scientific computing
- [ ] Other undergraduate coursework (please specify) ____________
- [ ] Graduate coursework (please specify) ____________

Powered by Qualtrics

S4 2020 Post-Survey

Preamble

The purpose of this survey is to help measure the effectiveness of the bootcamp. **We welcome responses from all of you even if you did not participate in the bootcamp, because it will help us gauge its utility.**

The survey consists of brief questions about your familiarity and comfort with certain math concepts, feedback about the bootcamp sessions and content, and 5 very short answer style math questions. **The survey may take you about 15 minutes to complete.**

The survey is not intended to be taken on mobile devices, which will not have optimized image formatting. If possible, we recommend completing it on a desktop or laptop computer.

General

Name

____

Email

____

Which course(s) are you planning on taking this semester?

- [ ] Thermodynamics and Statistical Mechanics (CHEM 220A)
- [ ] Advanced Quantum Mechanics (CHEM 221A)
- [ ] Quantum Mechanics (PHYS 221A)
- [ ] I don't know yet
- [ ] Other (please specify) __________

Which sessions did you attend in the bootcamp (Aug 6-12)?

- ☐ Integration and Differentiation (Aug 6)
- ☐ Methods and Approximations (Aug 6)
- ☐ Linear Algebra 1 (Aug 7)
- ☐ Linear Algebra 2 (Aug 7)
- ☐ Functions (Aug 10)
- ☐ Linear Algebra 3 (Aug 10)
- ☐ Differential Equations 1 (Aug 11)
- ☐ Differential Equations 2 (Aug 11)
- ☐ Probability (Aug 12)
- ☐ Questions and discussion (Aug 12)
- ☐ I did not attend any bootcamp sessions

Indicate the approximate number of hours you've spent engaging with the following bootcamp material after its conclusion.

	< 1 hour	1-3 hours	3-6 hours	> 6 hours
Written notes / slides	○	○	○	○
Suggested videos	○	○	○	○
Practice problems and solutions	○	○	○	○

How helpful have you found the following resources? (1 = very unhelpful, 5 = very helpful)

	0	1	2	3	4	5
Written notes / slides						
Suggested videos						
Practice problems and solutions						

Confidence

Indicate your comfort level with each of the following branches of mathematics (1 = very uncomfortable, 5 = very comfortable):

	1	2	3	4	5
Single variable integration	○	○	○	○	○
Multivariable integration	○	○	○	○	○
Single variable differentiation	○	○	○	○	○
Multivariable differentiation	○	○	○	○	○
Coordinate transformations (polar, spherical, Cartesian, etc.)	○	○	○	○	○
Solving differential equations	○	○	○	○	○
Approximating functions with series expansions	○	○	○	○	○
Decomposing a vector in a basis	○	○	○	○	○
Determining the eigenvalues and eigenvectors of a matrix	○	○	○	○	○
Using Dirac notation	○	○	○	○	○
Thinking about high-dimensional vector spaces	○	○	○	○	○
Counting and probability	○	○	○	○	○

Indicate your comfort level with each of the following activities as pertaining to math-related content (1 = very uncomfortable, 5 = very comfortable):

	1	2	3	4	5
Solving problems in a group	○	○	○	○	○
Asking professors or TAs/GSIs about material	○	○	○	○	○
Identifying relevant online or textbook resources	○	○	○	○	○

	1	2	3	4	5
Learning / working over zoom	○	○	○	○	○

Math preamble

The following 5 short answer style math questions will help us gauge your familiarity with certain types of math concepts and questions, and will help us determine the utility and effectiveness of the math bootcamp.

For each question, please write the short answer or the letter of the best provided answer. You may want to use some scratch paper to help you work out your answer. Please do your best to answer each question.

It is **still** totally okay if you are unable to answer all (or any!) of the following problems. We want to emphasize that solving arbitrary math problems with minimal context and warmup does not reflect your potential for being a successful student or researcher. **Regardless of what our imposter syndrome may tell us, all of us belong here, in this department.** The sole purpose of these questions is to understand the effectiveness of the bootcamp in building skills and confidence to answer these specific types of math problems.

Probability

Is $f(r) = \frac{4}{a^3} r^2 \exp(-2r/a)$ a valid probability distribution?

(a) Yes

(b) Yes, but only for $r > 0$

(c) No, it's not normalized

(d) No, $f(r)$ cannot be greater than 1

Evaluate the following integral: $I = \int_{-\infty}^{\infty} \exp(-\frac{x^2}{2\sigma^2})$

(a) $I = 1$

(b) $I = 1/\sqrt{2\pi\sigma^2}$

(c) $I = \sqrt{2\pi\sigma^2}$

(d) $I = \pi$

How many ways can n indistinguishable particles be placed in k distinguishable boxes?

(a) $\frac{n!}{k!}$

(b) $\binom{n}{k} = \frac{n!}{k!(n-k)!}$

(c) $\frac{n!}{(n-k)!}$

(d) $\binom{n+k-1}{k-1} = \binom{n+k-1}{n} = \frac{(n+k-1)!}{n!(k-1)!}$

Let $E(x)$ be a function describing the energy of a particle as a function of its position x. Which distribution would you use to evaluate the energy of a particle constrained to a certain position x_0?

(a) Gaussian

(b) Dirac delta

(c) Poisson

(d) Binomial

A random variable X has a mean of μ and variance of σ^2. Let another random variable $Y = 3X + 4$. What is the variance of Y?

How confident were you in your ability to answer this question?

- 1 Not at all confident
- 2
- 3 Somewhat confident
- 4
- 5 Very confident

Calculus

What is the shape of the region of xyz-space where $x+y+z \leq 1$ and $x, y, z \geq 0$?

(a) Cone

(b) Ellipsoid

(c) Paraboloid

(d) Rectangular Prism

(e) Tetrahedron

Suppose that $z = f(x(t), y(t))$ where $\left(\frac{\partial z}{\partial x}\right)_y = 3$, $\left(\frac{\partial z}{\partial y}\right)_x = 1$, $\frac{dx}{dt} = 7$, $\frac{dy}{dt} = 2$.

What is $\frac{dz}{dt}$?

What is the slope of $f(x, y) = 2x^3y + y$ in the y-direction at $(1, -1)$?

In a Taylor Series, what does the constant term represent?

When changing variables from Cartesian to spherical coordinates the volume differential $dx\, dy\, dz$ is replaced by $r^2 \sin\phi\, dr\, d\phi\, d\theta$. Explain the factor of r^2 and the factor of $\sin\phi$.

How confident were you in your ability to answer this question?

- 1 Not at all confident
- 2
- 3 Somewhat confident
- 4
- 5 Very confident

Differential Equations

The ODE $\frac{d^2y}{dx^2} = 0$ represents $y(x)$ as a

(a) Family of horizontal lines

(b) Family of vertical lines

(c) Family of straight lines with a fixed slope

(d) Set of arbitrary straight lines

What is the dimension of phase space for a particle trajectory in 3D?

Which integrating factor will make the ODE $(x \ln x)dy + ydx = 0$ exact?

(a) x

(b) $1/x$

(c) $1/\ln x$

What is the general solution of $\frac{d^2y}{dx^2} - 3\frac{dy}{dx} + 2y = 0$?

The infinite power series that will solve the ODE $\frac{dy}{dx} = 1 - xy$ is

(a) $y = 1 + x + \frac{x^2}{2!} + \frac{x^3}{3!} + \ldots$

(b) $y = 1 + \frac{x^2}{(2)(1)} + \frac{x^4}{(4)(3)} + \ldots$

(c) $y = x - \frac{x^3}{(3)(1)} + \frac{x^5}{(5)(3)(1)} - \ldots$

(d) $y = x - \frac{x^3}{3} + \frac{x^5}{5} - \ldots$

How confident were you in your ability to answer this question?

- 1 Not at all confident
- 2
- 3 Somewhat confident
- 4
- 5 Very confident

Functions

What is the sum of $1 - \frac{1}{2} + \frac{1}{4} - \frac{1}{8} + \ldots$?

The Fourier series expansion of $y = 1/(x - x^5)$ will have

(a) Only cosines

(b) Only sines

(c) Cosine coefficients = corresponding sine coefficients

(d) Cosine coefficients = − corresponding sine coefficients

The power series expansion for $\sinh x = \frac{e^x - e^{-x}}{2}$ is

(a) $1 + \frac{x^2}{2!} + \frac{x^4}{4!} + ...$

(b) $x + \frac{x^3}{3!} + \frac{x^5}{5!} + ...$

(c) $x - \frac{x^3}{3!} + \frac{x^5}{5!} - ...$

What are the diagonal elements of the double derivative operator d^2/dx^2 in this exponential space $\{1, e^{-x}, e^{-2x}, e^{-3x}, ...\}$?

The Fourier transform $g(\omega)$ of a function $f(t)$ is to be defined as

$$g(\omega) = \frac{1}{\sqrt{2\pi}} \int_{-\infty}^{\infty} f(t) e^{i\omega t} dt$$

Match the functions to their corresponding Fourier transforms in the following two figures.

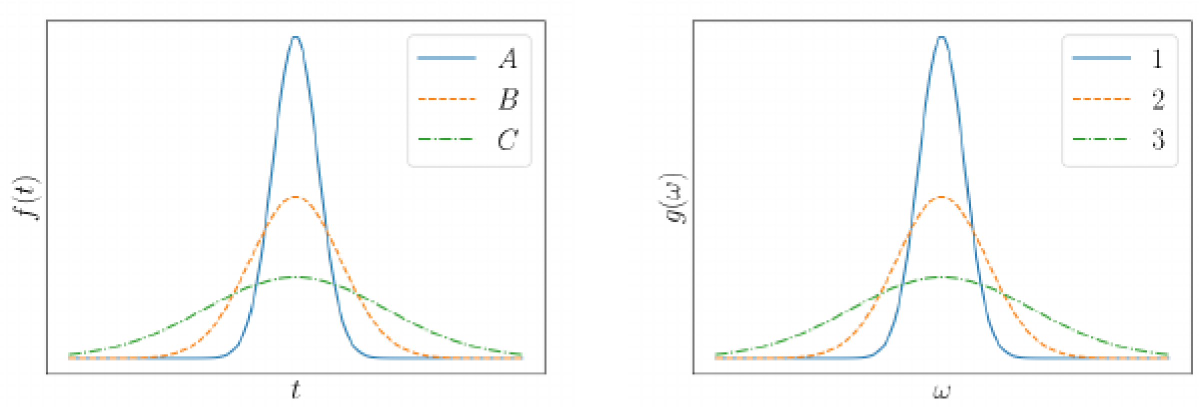

How confident were you in your ability to answer this question?

- 1 Not at all confident
- 2
- 3 Somewhat confident
- 4
- 5 Very confident

Linear Algebra

What is the dimension of the space spanned by the following two vectors?

$$\begin{bmatrix} 2 \\ 3 \\ 1 \end{bmatrix} \quad \begin{bmatrix} 1 \\ 0 \\ -1 \end{bmatrix}$$

What are the eigenvalues of the matrix $\mathbf{A}$?

$$\mathbf{A} = \begin{bmatrix} 4 & 2 & 0 \\ 1 & 5 & 0 \\ 0 & 0 & -3 \end{bmatrix}$$

Which of the following vectors is orthogonal to the vector $\mathbf{v}$? (Select all that apply)

$$\mathbf{v} = \begin{bmatrix} 2 \\ -3 \\ i \end{bmatrix}$$

$$\mathbf{a} = \begin{bmatrix} i \\ 0 \\ -2 \end{bmatrix}, \quad \mathbf{b} = \begin{bmatrix} 1 \\ 1 \\ i \end{bmatrix}, \quad \mathbf{c} = \begin{bmatrix} 1+i \\ 0 \\ 2 \end{bmatrix}, \quad \mathbf{d} = \begin{bmatrix} 3 \\ -2 \\ 1 \end{bmatrix}$$

- ☐ a
- ☐ b
- ☐ c
- ☐ d

Expressed in the basis $|a\rangle, |b\rangle, |c\rangle, |d\rangle$, the operator $\mathbf{A}$ has the following matrix representation:

$$\begin{bmatrix} 1 & 0 & -3 & 0 \\ 0 & -1 & 0 & 0 \\ 2 & 0 & -2 & 0 \\ 0 & 0 & 0 & 4 \end{bmatrix}.$$

Which (if any) of $|a\rangle, |b\rangle, |c\rangle$, and $|d\rangle$ are eigenvectors of $\mathbf{A}$? Select all that apply. (You do not need to explicitly solve for the eigensystem of $\mathbf{A}$ in solving this problem.)

(a) $|a\rangle$

(b) $|b\rangle$

(c) $|c\rangle$

(d) $|d\rangle$

- ☐ a

- [ ] b
- [ ] c
- [ ] d

Which of the following are linear operators? (Select all that apply)

(a) $\hat{A} = x$

(b) $\hat{B} = \frac{\partial}{\partial x}$

(c) $\hat{C} = \int_{-\infty}^{\infty} dx$

(d) $\hat{D} = \frac{\partial}{\partial x} x$

- [ ] A
- [ ] B
- [ ] C
- [ ] D

How confident were you in your ability to answer this question?

- ○ 1 Not at all confident
- ○ 2
- ○ 3 Somewhat confident
- ○ 4
- ○ 5 Very confident

General Feedback

Thank you for participating in the bootcamp and completing the survey. We would love to hear any feedback you have so that we can improve the bootcamp in future years.

Do not hesitate to reach out to us at ucbmathbootcamp@gmail.com if you have anything (questions, comments, etc) that you'd like to discuss!

Are there any topics you wish we had covered that we did not? Are there any topics you wish we had spent more or less time on?

Do you have any other feedback regarding the bootcamp -- either the zoom sessions or the related material?

Powered by Qualtrics

S5 2021 Post-Survey

Preamble

The purpose of this survey is to help measure the effectiveness of the bootcamp. **We welcome responses from all of you even if you did not participate in the bootcamp, because it will help us gauge its utility.**

The survey consists of brief questions about your familiarity and comfort with certain math concepts, feedback about the bootcamp sessions and content, and 5 very short answer style math questions. **The survey may take you about 15 minutes to complete.**

The survey is not intended to be taken on mobile devices, which will not have optimized image formatting. If possible, we recommend completing it on a desktop or laptop computer.

General

Name

[ ]

Email

[ ]

Which course(s) are you planning on taking this semester?

- [ ] Thermodynamics and Statistical Mechanics (CHEM 220A)
- [ ] Advanced Quantum Mechanics (CHEM 221A)
- [ ] Quantum Mechanics (PHYS 221A)
- [ ] I don't know yet
- [ ] Other (please specify) [ ]

Which sessions did you attend in the bootcamp (Aug 5-11)?

- ☐ Integration and Differentiation (Aug 5)
- ☐ Methods and Approximations (Aug 5)
- ☐ Python (Aug 6)
- ☐ Linear Algebra 1 (Aug 6)
- ☐ Linear Algebra 2 (Aug 9)
- ☐ Linear Algebra 3 (Aug 9)
- ☐ Fourier Analysis (Aug 10)
- ☐ Differential Equations 1 (Aug 10)
- ☐ Differential Equations 2 (Aug 11)
- ☐ Probability (Aug 11)
- ☐ I did not attend any bootcamp sessions

Indicate the approximate number of hours you've spent engaging with the following bootcamp material after its conclusion.

	< 1 hour	1-3 hours	3-6 hours	> 6 hours
Written notes / slides	○	○	○	○
Suggested videos	○	○	○	○
Practice problems and solutions	○	○	○	○

How helpful have you found the following resources? (1 = very unhelpful, 5 = very helpful)

	0	1	2	3	4	5
Written notes / slides						
Suggested videos						
Practice problems and solutions						

Confidence

Indicate your comfort level with each of the following branches of mathematics (1 = very uncomfortable, 5 = very comfortable):

	1	2	3	4	5
Single variable integration	○	○	○	○	○
Multivariable integration	○	○	○	○	○
Single variable differentiation	○	○	○	○	○
Multivariable differentiation	○	○	○	○	○
Coordinate transformations (polar, spherical, Cartesian, etc.)	○	○	○	○	○
Solving differential equations	○	○	○	○	○
Approximating functions with series expansions	○	○	○	○	○
Decomposing a vector in a basis	○	○	○	○	○
Determining the eigenvalues and eigenvectors of a matrix	○	○	○	○	○
Using Dirac notation	○	○	○	○	○
Thinking about high-dimensional vector spaces	○	○	○	○	○
Counting and probability	○	○	○	○	○
Writing or reading computer code	○	○	○	○	○

Indicate your comfort level with each of the following activities as pertaining to math-related content (1 = very uncomfortable, 5 = very comfortable):

	1	2	3	4	5
Solving problems in a group	○	○	○	○	○
Asking professors or TAs/GSIs about material	○	○	○	○	○

	1	2	3	4	5
Identifying relevant online or textbook resources	○	○	○	○	○
Learning / working over zoom	○	○	○	○	○

Math preamble

The following 5 short answer style math questions will help us gauge your familiarity with certain types of math concepts and questions, and will help us determine the utility and effectiveness of the math bootcamp.

For each question, please write the short answer or the letter of the best provided answer. You may want to use some scratch paper to help you work out your answer. Please do your best to answer each question.

It is **still** totally okay if you are unable to answer all (or any!) of the following problems. We want to emphasize that solving arbitrary math problems with minimal context and warmup does not reflect your potential for being a successful student or researcher. **Regardless of what our imposter syndrome may tell us, all of us belong here, in this department.** The sole purpose of these questions is to understand the effectiveness of the bootcamp in building skills and confidence to answer these specific types of math problems.

Probability

Is $f(r) = \frac{4}{a^3} r^2 \exp(-2r/a)$ a valid probability distribution?

(a) Yes

(b) Yes, but only for $r > 0$

(c) No, it's not normalized

(d) No, $f(r)$ cannot be greater than 1

Evaluate the following integral: $I = \int_{-\infty}^{\infty} \exp(-\frac{x^2}{2\sigma^2})$

(a) $I = 1$

(b) $I = 1/\sqrt{2\pi\sigma^2}$

(c) $I = \sqrt{2\pi\sigma^2}$

(d) $I = \pi$

How many ways can n indistinguishable particles be placed in k distinguishable boxes?

(a) $\frac{n!}{k!}$

(b) $\binom{n}{k} = \frac{n!}{k!(n-k)!}$

(c) $\frac{n!}{(n-k)!}$

(d) $\binom{n+k-1}{k-1} = \binom{n+k-1}{n} = \frac{(n+k-1)!}{n!(k-1)!}$

Let $E(x)$ be a function describing the energy of a particle as a function of its position x. Which distribution would you use to evaluate the energy of a particle constrained to a certain position x_0?

(a) Gaussian

(b) Dirac delta

(c) Poisson

(d) Binomial

A random variable X has a mean of μ and variance of σ^2. Let another random variable $Y = 3X + 4$. What is the variance of Y?

How confident were you in your ability to answer this question?

- 1 Not at all confident
- 2
- 3 Somewhat confident
- 4
- 5 Very confident

Calculus

What is the shape of the region of xyz-space where $x+y+z \leq 1$ and $x, y, z \geq 0$?

(a) Cone

(b) Ellipsoid

(c) Paraboloid

(d) Rectangular Prism

(e) Tetrahedron

Suppose that $z = f(x(t), y(t))$ where $\left(\frac{\partial z}{\partial x}\right)_y = 3$, $\left(\frac{\partial z}{\partial y}\right)_x = 1$, $\frac{dy}{dt} = 7$, $\frac{dx}{dt} = 2$. What is $\frac{dz}{dt}$?

What is the slope of $f(x, y) = 2x^3y + y$ in the y-direction at $(1, -1)$?

Where does the constant term in the Taylor expansion of a function come from?

When changing variables from Cartesian to spherical coordinates, the volume differential $dx\,dy\,dz$ is replaced by $r^2 \sin\phi\, dr\, d\phi\, d\theta$. Why do we include the factors of r^2 and $\sin\theta$?

How confident were you in your ability to answer this question?

- 1 Not at all confident
- 2
- 3 Somewhat confident
- 4
- 5 Very confident

Differential Equations

The ODE $\frac{d^2y}{dx^2} = 0$ represents $y(x)$ as a

(a) Family of horizontal lines

(b) Family of vertical lines

(c) Family of straight lines with a fixed slope

(d) Set of arbitrary straight lines

What is the dimension of phase space for a particle trajectory in 3D?

Which integrating factor will make the ODE $(x \ln x)dy + ydx = 0$ exact?

(a) x

(b) $1/x$

(c) $1/\ln x$

What is the general solution of $\frac{d^2y}{dx^2} - 3\frac{dy}{dx} + 2y = 0$?

The infinite power series that will solve the ODE $\frac{dy}{dx} = 1 - xy$ is

(a) $y = 1 + x + \frac{x^2}{2!} + \frac{x^3}{3!} + ...$

(b) $y = 1 + \frac{x^2}{(2)(1)} + \frac{x^4}{(4)(3)} + ...$

(c) $y = x - \frac{x^3}{(3)(1)} + \frac{x^5}{(5)(3)(1)} - ...$

(d) $y = x - \frac{x^3}{3} + \frac{x^5}{5} - ...$

How confident were you in your ability to answer this question?

- 1 Not at all confident
- 2
- 3 Somewhat confident
- 4
- 5 Very confident

Functions

What is the sum of $1 - \frac{1}{2} + \frac{1}{4} - \frac{1}{8} + ...$?

The Fourier series expansion of $y = 1/(x - x^5)$ will have

(a) Only cosines

(b) Only sines

(c) Cosine coefficients = corresponding sine coefficients

(d) Cosine coefficients = − corresponding sine coefficients

The power series expansion for $\sinh x = \frac{e^x - e^{-x}}{2}$ is

(a) $1 + \frac{x^2}{2!} + \frac{x^4}{4!} + ...$

(b) $x + \frac{x^3}{3!} + \frac{x^5}{5!} + ...$

(c) $x - \frac{x^3}{3!} + \frac{x^5}{5!} - ...$

What are the diagonal elements of the double derivative operator d^2/dx^2 in this exponential space $\{1, e^{-x}, e^{-2x}, e^{-3x}, ...\}$?

The Fourier transform $g(\omega)$ of a function $f(t)$ is to be defined as

$$g(\omega) = \frac{1}{\sqrt{2\pi}} \int_{-\infty}^{\infty} f(t) e^{i\omega t} dt$$

Match the functions to their corresponding Fourier transforms in the following two figures.

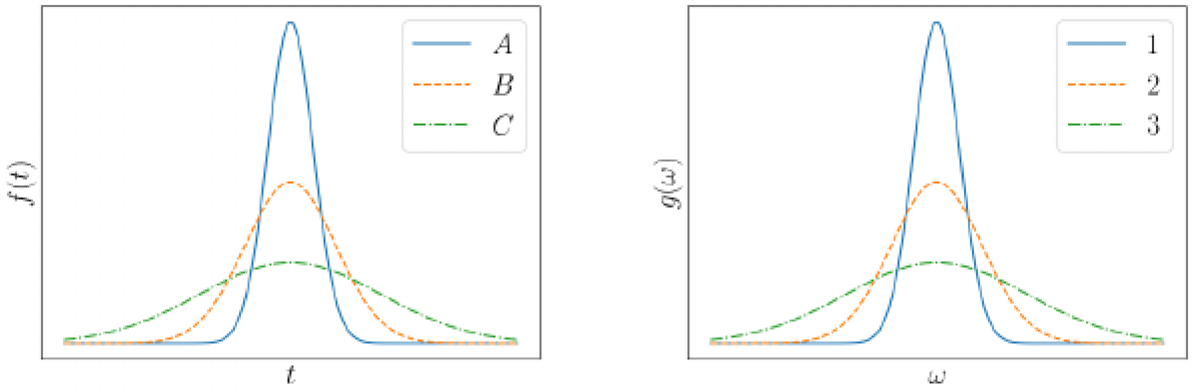

How confident were you in your ability to answer this question?

- 1 Not at all confident
- 2
- 3 Somewhat confident
- 4
- 5 Very confident

Linear Algebra

What is the dimension of the space spanned by the following two vectors?

$$\begin{bmatrix} 2 \\ 3 \\ 1 \end{bmatrix} \quad \begin{bmatrix} 1 \\ 0 \\ -1 \end{bmatrix}$$

What are the eigenvalues of the matrix $\mathbf{A}$?

$$\mathbf{A} = \begin{bmatrix} 4 & 2 & 0 \\ 1 & 5 & 0 \\ 0 & 0 & -3 \end{bmatrix}$$

Which of the following vectors is orthogonal to the vector $\mathbf{v}$? (Select all that apply)

$$\mathbf{v} = \begin{bmatrix} 2 \\ -3 \\ i \end{bmatrix}$$

$$\mathbf{a} = \begin{bmatrix} i \\ 0 \\ -2 \end{bmatrix}, \quad \mathbf{b} = \begin{bmatrix} 1 \\ 1 \\ i \end{bmatrix}, \quad \mathbf{c} = \begin{bmatrix} 1+i \\ 0 \\ 2 \end{bmatrix}, \quad \mathbf{d} = \begin{bmatrix} 3 \\ -2 \\ 1 \end{bmatrix}$$

- ☐ a
- ☐ b
- ☐ c
- ☐ d

Expressed in the basis $|a\rangle, |b\rangle, |c\rangle, |d\rangle$, the operator $\mathbf{A}$ has the following matrix representation:

$$\begin{bmatrix} 1 & 0 & -3 & 0 \\ 0 & -1 & 0 & 0 \\ 2 & 0 & -2 & 0 \\ 0 & 0 & 0 & 4 \end{bmatrix}.$$

Which (if any) of $|a\rangle, |b\rangle, |c\rangle$, and $|d\rangle$ are eigenvectors of $\mathbf{A}$? Select all that apply. (You do not need to explicitly solve for the eigensystem of $\mathbf{A}$ in solving this problem.)

(a) $|a\rangle$

(b) $|b\rangle$

(c) $|c\rangle$

(d) $|d\rangle$

- ☐ a

- [ ] b
- [ ] c
- [ ] d

Which of the following are linear operators? (Select all that apply)

(a) $\hat{A} = x$

(b) $\hat{B} = \frac{\partial}{\partial x}$

(c) $\hat{C} = \int_{-\infty}^{\infty} dx$

(d) $\hat{D} = \frac{\partial}{\partial x} x$

- [ ] A
- [ ] B
- [ ] C
- [ ] D

How confident were you in your ability to answer this question?

- ○ 1 Not at all confident
- ○ 2
- ○ 3 Somewhat confident
- ○ 4
- ○ 5 Very confident

General Feedback

Thank you for participating in the bootcamp and completing the survey. We would love to hear any feedback you have so that we can improve the bootcamp in future years.

Do not hesitate to reach out to us at ucbmathbootcamp@gmail.com if you have anything (questions, comments, etc) that you'd like to discuss!

Are there any topics you wish we had covered that we did not? Are there any topics you wish we had spent more or less time on?

Do you have any other feedback regarding the bootcamp -- either the zoom sessions or the related material?

Powered by Qualtrics

S6 2020 Final Survey

Preamble

The purpose of this survey is to help measure the effectiveness of the bootcamp. **We welcome responses from all of you even if you did not participate in the bootcamp, because it will help us gauge its utility.**

The survey consists of brief questions about your familiarity and comfort with certain math concepts and general feedback about the bootcamp. **The survey may take you about 5-10 minutes to complete.**

The survey is not intended to be taken on mobile devices, which will not have optimized image formatting. If possible, we recommend completing it on a desktop or laptop computer.

General

Name

Email

Which course(s) did you take in this Fall semester?

- ☐ Thermodynamics and Statistical Mechanics (CHEM 220A)
- ☐ Advanced Quantum Mechanics (CHEM 221A)
- ☐ Quantum Mechanics (PHYS 221A)
- ☐ Other (please specify)

Indicate the approximate number of hours you've spent engaging with the following bootcamp material after its conclusion.

	< 1 hour	1-3 hours	3-6 hours	> 6 hours
Written notes / slides	○	○	○	○
Suggested videos	○	○	○	○
Practice problems and solutions	○	○	○	○

How helpful have you found the following resources? (1 = very unhelpful, 5 = very helpful)

	0	1	2	3	4	5
Written notes / slides						
Suggested videos						
Practice problems and solutions						

Confidence

Indicate your comfort level with each of the following branches of mathematics (1 = very uncomfortable, 5 = very comfortable):

	1	2	3	4	5
Single variable integration	○	○	○	○	○
Multivariable integration	○	○	○	○	○
Single variable differentiation	○	○	○	○	○
Multivariable differentiation	○	○	○	○	○
Coordinate transformations (polar, spherical, Cartesian, etc.)	○	○	○	○	○
Solving differential equations	○	○	○	○	○
Approximating functions with series expansions	○	○	○	○	○

	1	2	3	4	5
Decomposing a vector in a basis	○	○	○	○	○
Determining the eigenvalues and eigenvectors of a matrix	○	○	○	○	○
Using Dirac notation	○	○	○	○	○
Thinking about high-dimensional vector spaces	○	○	○	○	○
Counting and probability	○	○	○	○	○

Indicate your comfort level with each of the following activities as pertaining to math-related content (1 = very uncomfortable, 5 = very comfortable):

	1	2	3	4	5
Solving problems in a group	○	○	○	○	○
Asking professors or TAs/GSIs about material	○	○	○	○	○
Identifying relevant online or textbook resources	○	○	○	○	○
Learning / working over Zoom	○	○	○	○	○

The following two questions ask you to self-report your Fall grades. They are optional and your responses will only be analyzed in a de-identified form.

If you took Chem 220A, what was your final letter grade in the course?

If you took Chem 221A, what was your final letter grade in the course?

General Feedback

Do you have any thoughts or reflections, either positive or negative, about how participating in the math bootcamp has affected your experience in graduate school, both as it pertains to your coursework and generally?

Are there any topics you wish we had covered that we did not? Are there any topics you wish we had spent more or less time on?

Do you have any other feedback regarding the bootcamp—either the zoom sessions or the related material?

Final Page

Thank you for participating in the bootcamp and completing the survey. We would love to hear any feedback you have so that we can improve the bootcamp in future years.

Do not hesitate to reach out to us at ucbmathbootcamp@gmail.com if you have anything (questions, comments, etc.) that you'd like to discuss!

Powered by Qualtrics

S7 2021 Final Survey

Preamble

The purpose of this survey is to help measure the effectiveness of the bootcamp. **We welcome responses from all of you even if you did not participate in the bootcamp, because it will help us gauge its utility.**

The survey consists of brief questions about your familiarity and comfort with certain math concepts and general feedback about the bootcamp. **The survey may take you about 5-10 minutes to complete.**

General

Name

[ ]

Email

[ ]

Which course(s) did you take in this Fall semester?

- ☐ Thermodynamics and Statistical Mechanics (CHEM 220A)
- ☐ Advanced Quantum Mechanics (CHEM 221A)
- ☐ Quantum Mechanics (PHYS 221A)
- ☐ Other (please specify) [ ]

Indicate the approximate number of hours you've spent engaging with the following bootcamp material after its conclusion.

	< 1 hour	1-3 hours	3-6 hours	> 6 hours

	< 1 hour	1-3 hours	3-6 hours	> 6 hours
Written notes / slides	○	○	○	○
Suggested videos	○	○	○	○
Practice problems and solutions	○	○	○	○

How helpful have you found the following resources? (1 = very unhelpful, 5 = very helpful)

	0	1	2	3	4	5
Written notes / slides						
Suggested videos						
Practice problems and solutions						

General Feedback

How did participating in the math bootcamp affect your transition into and experience in graduate school coursework?

How did participating in the bootcamp affect your ability to find peers to work with during your first semester? Did it affect your sense of belonging in the College of Chemistry?

Are there any topics you wish we had covered that we did not? Are there any topics you wish we had spent more or less time on?

Do you have any thoughts on how we can encourage more collaboration between students in the future?

Do you have any other feedback regarding the bootcamp—either the zoom sessions or the related material?

Confidence

Indicate your comfort level with each of the following branches of mathematics (1 = very uncomfortable, 5 = very comfortable):

	1	2	3	4	5
Single variable integration	○	○	○	○	○
Multivariable integration	○	○	○	○	○
Single variable differentiation	○	○	○	○	○
Multivariable differentiation	○	○	○	○	○
Coordinate transformations (polar, spherical, Cartesian, etc.)	○	○	○	○	○
Solving differential equations	○	○	○	○	○
Approximating functions with series expansions	○	○	○	○	○
Decomposing a vector in a basis	○	○	○	○	○
Determining the eigenvalues and eigenvectors of a matrix	○	○	○	○	○
Using Dirac notation	○	○	○	○	○
Thinking about high-dimensional vector spaces	○	○	○	○	○
Counting and probability	○	○	○	○	○
Writing or reading computer code	○	○	○	○	○

Indicate your comfort level with each of the following activities as pertaining to math-related content (1 = very uncomfortable, 5 = very comfortable):

	1	2	3	4	5
Solving problems in a group	○	○	○	○	○
Asking professors or TAs/GSIs about material	○	○	○	○	○
Identifying relevant online or textbook resources	○	○	○	○	○

The following two questions ask you to self-report your Fall grades. They are optional and your responses will only be analyzed in a de-identified form.

If you took Chem 220A, what was your final letter grade in the course?

If you took Chem 221A, what was your final letter grade in the course?

Final Page

Thank you for participating in the bootcamp and completing the survey. We would love to hear any feedback you have so that we can improve the bootcamp in future years.

Do not hesitate to reach out to us at ucbmathbootcamp@gmail.com if you have anything (questions, comments, etc.) that you'd like to discuss!

Powered by Qualtrics

S8 2020 Professor Interview Questions

Interview Protocol 2020 - Professor

1. Was there a noticeable difference in student performance related to solving the homework assignments?

2. Was a change in how students approached solving homework problems that you noticed during discussion or office hour sections?

3. Was there a change in student enthusiasm about discussing math problem solving online over zoom?

4. Was there a change in students' willingness to participate in discussions on solving math problems?

5. Are there math-related skills or material that students particularly struggled with? Skills or material that students did well with, despite your expectations? (Follow up: did we cover those topics in the bootcamp? Should we spend more or less time on those?)

6. Did you notice any change in homework/midterm/final grade distributions and the mean of those distributions, compared to the previous year (2019)?

7. Do you have any reflections or feedback regarding the bootcamp based off of your experience teaching this course?

S9 2021 Professor Interview Questions

Interview Protocol 2021 - Professor

[Note: for changes, it's good to ask about changes relative to both 2020 and 2019 and prior. This year there are bound to be changes as the Bootcamp and classes were held in person.]

1. Was there a noticeable difference in student performance related to solving the homework assignments?

2. Was there a change in how students approached solving homework problems that you noticed during discussion or office hour sections?

3. [No longer relevant, as Fall 2021 classes were held in person, not over zoom.] Was there a change in student enthusiasm about discussing math problem solving online over zoom?

4. Was there a change in students' willingness to participate in discussions on solving math problems?

5. Are there math-related skills or material that students particularly struggled with this year? Skills or material that students did well with, despite your expectations? (Follow up: did we cover those topics in the bootcamp? Should we spend more or less time on those?)

6. Did you notice any change in homework/midterm/final grade distributions and the mean of those distributions, compared to the previous years (2020, 2019)?

7. Do you have any reflections or feedback regarding the bootcamp based on your experience teaching this course?

S10 2020 GSI Interview Questions

Interview Protocol 2020 - GSI

1. Towards the beginning of semester, was there a noticeable effect of the bootcamp on student performance related to solving the homework assignments or approaching homework problems that you noticed during discussion or office hour sections?

2. Was there any effect of the bootcamp on students' enthusiasm to participate in collaborative discussions on solving math problems on Zoom?

3. Are there math-related skills or material that students particularly struggled with? Skills or material that students did well with, despite your expectations? Do you recommend we covered any specific materials?

4. Do you have any reflections or feedback regarding the bootcamp based off of your experience teaching this course? What is and what isn't helpful?

S11 2021 GSI Interview Questions

Interview Protocol 2021 - GSI

1. Towards the beginning of semester, was there a noticeable effect of the bootcamp on student performance related to solving the homework assignments or approaching homework problems that you noticed during discussion or office hour sections?

2. [No longer relevant, as Fall 2021 classes were held in person, not over zoom.] Was there any effect of the bootcamp on students' enthusiasm to participate in collaborative discussions on solving math problems on Zoom?

3. Are there math-related skills or material that students particularly struggled with? Skills or material that students did well with, despite your expectations? Do you recommend we cover any specific materials?

4. Do you have any reflections or feedback regarding the bootcamp based on your experience teaching this course? What is and what isn't helpful?
